# Short-Chain Fatty Acids Modulate Metabolic Pathways and Membrane Lipids in *Prevotella bryantii* B_1_4

**DOI:** 10.3390/proteomes8040028

**Published:** 2020-10-16

**Authors:** Andrej Trautmann, Lena Schleicher, Simon Deusch, Jochem Gätgens, Julia Steuber, Jana Seifert

**Affiliations:** 1 Institute of Animal Science, University of Hohenheim, 70599 Stuttgart, Germany; Andrej.Trautmann@uni-hohenheim.de (A.T.); deusch@uni-hohenheim.de (S.D.); 2 Institute of Biology, University of Hohenheim, 70599 Stuttgart, Germany; lena.schleicher@uni-hohenheim.de (L.S.); julia.steuber@uni-hohenheim.de (J.S.); 3 Institute of Bio- and Geosciences, IBG-1: Biotechnology, Forschungszentrum Jülich, 52425 Jülich, Germany; j.gaetgens@fz-juelich.de

**Keywords:** short-chain fatty acids, long-chain fatty acids, branched-chain amino acids proteome, *Prevotella bryantii* B_1_4, lipid membrane, outer membrane proteins

## Abstract

Short-chain fatty acids (SCFAs) are bacterial products that are known to be used as energy sources in eukaryotic hosts, whereas their role in the metabolism of intestinal microbes is rarely explored. In the present study, acetic, propionic, butyric, isobutyric, valeric, and isovaleric acid, respectively, were added to a newly defined medium containing *Prevotella bryantii* B_1_4 cells. After 8 h and 24 h, optical density, pH and SCFA concentrations were measured. Long-chain fatty acid (LCFA) profiles of the bacterial cells were analyzed via gas chromatography-time of flight-mass spectrometry (GC-ToF MS) and proteins were quantified using a mass spectrometry-based, label-free approach. Cultures supplemented with single SCFAs revealed different growth behavior. Structural features of the respective SCFAs were identified in the LCFA profiles, which suggests incorporation into the bacterial membranes. The proteomes of cultures supplemented with acetic and valeric acid differed by an increased abundance of outer membrane proteins. The proteome of the isovaleric acid supplementation showed an increase of proteins in the amino acid metabolism. Our findings indicate a possible interaction between SCFAs, the lipid membrane composition, the abundance of outer membrane proteins, and a modulation of branched chain amino acid biosynthesis by isovaleric acid.

## 1. Introduction

Short-chain fatty acids (SCFAs) are formed by the microbial turnover of plant-derived materials in the rumen and serve as major energy source for animals [1]. In addition to the utilization of SCFAs by animals, they are also required for the growth of certain microorganisms like *Prevotella bryantii* B_1_4 [2], formerly named *Bacteroides ruminicola* or *Prevotella ruminicola* subsp. *brevis*. Copious SCFA-dependent microorganisms inhabit the gastrointestinal system of animals. The growth of various other bacterial species such as *Ruminococcus albus, Porphyromonas gingivalis* and *Fusobacterium prausnitzii* also require SCFAs [2,3,4]. Some members of the *Prevotellaceae* family such as *P. gingivalis, P. denticola*, or *P. intermedia* are opportunistic pathogens and likewise rely on host derived compounds [5,6]. *P. bryantii* B_1_4 on the other hand, is commonly found in the rumen and requires strictly anoxic conditions as well as carbon dioxide, heme, and vitamin K for growth promotion [7,8]. These conditions are usually met by adding rumen fluid to the respective culture media. The SCFAs in rumen fluid are mainly acetic (~50 mM), propionic (~20 mM), and butyric acid (~10 mM) and contain further isoforms of valeric acid (0.5–2 mM), which are well-known to be important for the ruminant metabolism [9]. For the host cells, SCFA are seen as a source of energy, a signal molecule, or a participating molecule in fatty acid and vitamin B biosynthesis [10,11,12]. Little is known about the role of SCFAs for prokaryotic cells wherefore similar functions are expected to be relevant for *P. bryantii* B_1_4. The use of SCFAs as a C-source can be implemented through coenzyme A attachment and subsequent oxidation. Regarding the anabolic way of the lipid metabolism, SCFAs can be utilized as precursor molecules for long-chain fatty acids (LCFAs). Fatty acid elongation is usually performed by transferring malonyl-CoA to acetyl-CoA by the catalytic activity of the 3-oxoacyl acyl-carrier-protein synthase. Instead of the acetyl-CoA, a SCFA-CoA can be transferred to malonyl-CoA [13]. Thereby, the SCFA-CoA forms the primer molecule and should be detected at the tail of the LCFA. It is also known that branched-chain amino acids (BCAAs) are converted for example into isovaleric acid or 2-methylbutyric acid [14,15]. It has been described that the amino group of BCAAs was first freed by the aminotransferase and second reduced to 2-oxoacids via the oxidoreductase [14]. A reverse reaction from branched SCFAs to BCAA was not proven yet but the reaction would be theoretically possible via an active incorporation by a ferredoxin oxidoreductase (EC 1.2.7.7).

In order to understand the fate of SCFA in *P. bryantii* B_1_4 cells, the present study established a growth medium enabling the growth of the bacteria on single SCFAs. This elucidates the mode of action of single SCFAs on growth behavior, consumption, and production of SCFAs, as well as on the assembly of membranes (LCFAs). Additionally, the effects of SCFA on overall metabolic routes and cellular mechanisms were analyzed by label-free quantification of the proteome.

## 2. Materials and Methods 

### 2.1. Cultivation in Medium M2-A for Proteome Analysis

*P. bryantii* B_1_4 (DSM 11371) was cultivated in Hungate tubes under anaerobic conditions. Composition of the medium (M2-A) was derived from the complex M2 medium [16], which is based on rumen fluid and yeast extract. In M2-A media rumen fluid and yeast extract were replaced by a vitamin solution (Table S1) and SCFAs or without SCFA (control) as described in Table S2 [8,17]. Media M2-B contained only glucose and a mixture of all used SCFAs in various concentrations (Table S2). Resazurin, minerals, sugars, and tryptic digested casein were dissolved, heated, and gassed with CO_2_, followed by addition of cysteine. After cooling down while being gassed continuously, the pH was adjusted to 6.8 by using 1 M NaOH. In the case of cultivations with single SCFAs, 15 mM of the respective acid was added to each Hungate tube. The filter sterilized vitamin solution was added by injection to the medium in the autoclaved Hungate tubes containing a final volume of 7.1 mL. The following SCFAs were used for cultivating: acetic acid (Acet; VWR), propionic acid (Prop; Carl Roth), n-butyric acid (But; Merck), isobutyric acid (iBut; Merck), n-valeric acid (Val; Merck) and isovaleric acid (iVal; Merck). *P. bryantii* B_1_4 cells from frozen glycerol stocks were first cultured using M2 medium. Concentrations of SCFA in rumen fluid usually depend on the feed and range from 0.5–50 mM [9]. The average of the sum of SCFAs was around 15 mM, which was therefore used in the present experiments.

Growing cells of the M2 medium (OD = 1.7–2.0) were used to inoculate the M2-A media (4% *v*/*v*) to an initial OD of 0.1. To assess successful growth (OD > 1.0) on M2-A media and to eliminate potential remains of the M2 medium, cells were transferred to fresh M2-A media two times. Each single SCFA culture was incubated in triplicates at 39 °C in a static water bath for 8 h to achieve a sufficient cell density. M2-A medium without SCFAs supplementation revealed no growth within 8 h after the second transfer. Cell growth was followed hourly by non-invasive optical density (OD) measurements in the Hungate tubes (λ = 600 nm; Bio Genesys™ 10, Thermo Fisher Scientific, Waltham, MA, USA). The pH was determined at the last time point of each growth experiment (pH meter, FE20, Mettler Toledo, Columbus, OH, USA). Cell pellets obtained by centrifugation at 4 °C with 10,000× *g* for 10 min were stored at −80 °C. Aliquots of the supernatant were stored at −20 °C for subsequent GC coupled to flame ionization detector (FID)-based SCFA quantification. 

### 2.2. Cultivation for Long-Chain Fatty Acid Determination

A cultivation volume of 100 mL in serum bottles containing M2-A medium was used to investigate the incorporation of SCFA into the long-chain fatty acids (LCFAs). Cells of two times transferred Hungate cultures were used for inoculation (7% *v*/*v*). Cells were incubated for 8 h or 24 h in order to observe changes in LCFA composition and SCFA depletion. The optical densities and pH were measured as previously described. Aliquots of the culture supernatants were stored at −20 °C for subsequent SCFA quantification. The cell pellets were stored at −80 °C for subsequent LCFA analyses.

### 2.3. Glucose Based Medium M2-B

The M2-A medium was altered by removing sodium lactate, maltose and cellobiose to establish medium M2-B. Except the adjustment from 2 g/L to 4 g/L of glucose and utilization of a SCFA mixture, the preparation of medium M2-B remained identical, as described above. Cultivation characteristics of *P. bryantii* B_1_4 in M2-B medium were equal to the M2-A media. A two-liter culture was used to allow frequent sampling of the culture. The 2-L culture was inoculated with 10% (*v*/*v*) and agitated with 100 rpm by a magnetic stir bar at 39 °C for 7 h. Samples were taken to determine OD, pH and glucose concentration. OD was analyzed photometrically in cuvettes (λ = 600 nm; Bio Genesys™ 10, Thermo Fisher Scientific). The pH was measured as previously described, and glucose concentration was determined down to the millimolar range using an enzyme kit (BioAnalysis D-Glucose, R-Biopharm, Darmstadt, Germany). 

### 2.4. Short-Chain Fatty Acid Determination

Determination of SCFAs was conducted using aliquots of the supernatants. The aliquots were thawed, vacuum distillated, and analyzed by a gas chromatograph (GC Hewlett-Packard 6890; Agilent, Santa Clara, CA, USA) equipped with a fused silica capillary column (HP-FFAP, 25 m × 0.32 mm, film thickness 0.5 µm HP 7683; Agilent) and FID with autosampler as described previously [18]. As internal standard, 80 mM of 2-methylvaleric acid in 50% formic acid was added to one aliquot for calibration. 

### 2.5. Long-Chain Fatty Acid Determination

Cell pellets were thawed on ice and washed twice using a 2.7% (w/v) NaCl solution. Cells were lyophilized (Gamma 1–20, Christ, Göttingen Germany) and homogenized, and 10 mg of each sample were mixed with 1.7 mL acidic methanol (10% *v*/*v* sulfuric acid) for in-situ transesterification at 60 °C and 750 rpm for 4 h in Pyrex glass tubes. After cooling down, 300 µL of desalted water was added. For methyl ester extraction, 1200 µL of n-heptane (HPLC grade, Chemsolute Th., Geyer, Germany) were added followed by proper vortexing for 20 s. The n-heptane phase was separated and stored at −20 °C until analysis. 

Methyl ester derivatized LCFAs were separated by a 30 m Agilent EZ-Guard VF-5ms + 10 m guard column (Agilent, Waldbronn, Germany) connected to an Agilent 6890N gas chromatograph (Agilent, Waldbronn, Germany). Compounds were analyzed by a Waters Micromass GCT Premier high-resolution time of flight mass spectrometer (Waters, Eschborn, Germany). Controlling was performed by the software MassLynx 4.1 (Waters, Eschborn, Germany), and injections were conducted by a Gerstel MPS 2 (Gerstel, Mülheim ad Ruhr, Germany) with Maestro software (Gerstel GmbH & Co. KG, Mühlheim an der Ruhr, Germany). Samples of 1 µL were injected into a split/splitless injector at 280 °C using altered split modes. A constant helium flow of 1 mL/min was applied. The GC temperature started at 60 °C for 2 min and increased by 12 °C/min up to a final temperature of 300 °C with a hold time of 8 min. Total runtime was 30 min. At 300 °C molecules were transferred into the TOF-MS, which operated in positive electron impact (EI)^+^ mode at an electron energy of 70 eV. The temperature at the ionization source was set to 180 °C. Calibration was tuned by Heptacosa (heptacosafluoro-tributylamine) fragment patterns. Accurate masses were corrected to a single point lockmass of chloro-pentafluoro-benzene (CPFB) as an external reference (*m*/*z* = 201.9609). Data acquisition was performed in centroid mode with a scan rate of 10 scans per second.

For the identification of known metabolites, a baseline noise subtracted fragment pattern was used and compared to the in-house database JuPoD and the commercial database NIST. Unknown peaks were identified by a structural combination of elemental compositions and verified by virtual derivatization and fragmentation of the predicted structure [19].

The illustration and comparison of LCFAs by the parameters of even or odd chain length and the structuring of linear, iso, or anteiso forms were similarly run as reported in former studies [20,21]. Quantified peak areas of LCFAs were summarized and represented in percentile contribution, which were used for Pearson correlation with the acidification (pH) and displaying it as the correlation coefficient (R). 

### 2.6. Protein Extraction

Protein extraction for subsequent LC-ESI-MS/MS analyses was carried out as described by Deusch, S., et al. [22]. Thawed cell pellets were kept on ice during the whole procedure. Pellets were washed twice in 300 µL wash buffer (50 mM Tris/HCl, pH 7.5, 0.1 mg/mL chloramphenicol, 1 mM PMSF) by vortexing and centrifugation at 10,000× *g*, 4 °C for 10 min. Pellets were resuspended in 50 µL wash buffer, and 50 µL extraction buffer (50 mM Tris/HCl, pH 7.5, 1% SDS) were added, followed by incubation at 1400 rpm and 60 °C for 10 min. Subsequently, 950 µL of a Benzonase solution (50 mM Tris/HCl, pH 7.5, 1 µL/mL Benzonase (Merck, Novagen, Germany), 0.1 mg/mL MgCl_2_, 1 mM PMSF) were added to the cells, followed by six times of ultra-sonication (UP 50H, Hielscher; 1 min at 60% amplitude and 0.5 per cycle). The samples were incubated at 37 °C and 1400 rpm for 10 min. Finally, the samples were centrifuged at 10,000× *g* and 4 °C for 10 min. The supernatants containing the extracted proteins were stored at −20 °C until further processing. Protein concentration was determined using the Bradford assay [23]. 

### 2.7. In Gel Protein Digestion

A five-fold volume of ice-cold acetone was added to 100 µg of protein for a 30 min precipitation. The samples were centrifuged for 10 min at 10,000× *g* and 4 °C, the supernatant was removed, and the protein pellet was dried at RT by vacuum centrifugation (Eppendorf concentrator, Hamburg, Germany). Protein pellets were resolved in 30 µL Laemmli buffer and loaded onto a sodium-dedocyl-sulfate (SDS)-polyacrylamide gel electrophoresis (PAGE, 4% stacking gel, 12% separation gel). The gels were run for 100 min at 40 mA. Each sample representing a separation gel lane of 1.0 cm length was cut out and subjected to overnight in-gel trypsin digestion (Promega, Madison, WI, USA) as described by Jehmlich, N., et al. [24]. Obtained peptides were cleaned by filtering through Stage-Tips with five layers of Empore™ SPE disks (3M Deutschland GmbH, Neuss, Germany) with a 47 mm diameter, 0.5 mm thickness, and C18 material [25]. The obtained peptides were dried and suspended in 0.1% formic acid before LC-ESI-MS/MS measurement. 

### 2.8. LC-ESI-MS/MS

The peptides were separated by an EASY-nLC1000 (Thermo Scientific, Dreieich, Germany) containing an EASY-Spray column PepMap RSLC (particle size 2 µm, 25 cm × 75 µm, C18). A sample volume of 4 µL was injected and liquid chromatography was run with a flow of 0.25 µL/min. The mobile phase consisted of solvent A (0.1% formic acid) and solvent B (80% acetonitrile, 0.1% formic acid). The gradient of solvent B (2-10-22-45-95%) was run over a time of 260 min (R_t_: 1-100-180-235-245 min). The peptides were ionized via electron spray ionization (ESI) before entering the Q-Exactive Plus mass spectrometer (Thermo Scientific) with a survey spectrum range from 300–1600 *m*/*z* in the Orbitrap mass analyzer and a resolution of 70,000. The second fragmentation was performed by high-energy collisional dissociation (HCD) with a resolution of 17,500. The 10 most abundant peptide precursors in the linear ion trap were generated by data dependent tandem mass spectra. Internal calibration was performed for all samples using lock-mass ions from ambient air as demonstrated earlier [26].

### 2.9. Data Analysis for Proteomics

Mass spectrometric (MS) data was analyzed by the software MaxQuant (v 1.6.2.6) [27]. Proteins were determined by searching peptide sequences from tandem MS data against the UniProtKB database of *P. bryantii* B_1_4 (3776 protein entries, 2 July 2019). The identification and label-free quantification (LFQ) considered only proteins with two or more unique peptide identifications. The options re-quantification and “match between runs” were enabled with a match time window of 0.7 min and an alignment time window of 20 min. Oxidation of methionine was set as variable modification and the carbamidomethylation of cysteine as fixed modification. Regarding tryptic digestion, a maximum of two missed cleavages were accepted. The peptides and proteins were filtered using a false discovery rate (FDR) of <1%. The MS data and MaxQuant output have been uploaded to the ProteomeXchange Consortium via PRIDE with the dataset identifier PXD016407 [28].

EggNOG and Ghost Koala were used for functional classification of quantified proteins to retrieve cluster of orthologous groups (COG) and the KEGG Orthology (KO) identifier [29,30]. KO assignments from both algorithms were combined prioritizing Ghost Koala. The LFQ values (including zeros) were implemented into the statistical software Primer 6 (v 6.1.16) and Permanova (v 1.0.6) (both PRIMER-E, Plymouth Marine Laboratory, Plymouth, UK) to generate PCO plots by standardizing them by the total and applying the Bray Curtis similarity test. LFQ values (including zeros) were used for SIMPER analysis and were cut-off when contribution was below 1%. The *p*-values for the LFQ proteins in the PCO plot were calculated by PERMANOVA analysis (9999 permutations) using the Monte Carlo correction of *p*-values. A protein was defined as present when least two of three replicates displayed an LFQ-value above zero. Non-quantified proteins can still be present but may be beyond the detection range of the mass spectrometer or MaxQuant. The abundances of core proteins in different metabolic pathways were used to compare incubation conditions. Abundance changes were determined by calculating the fold change of protein in condition with respect to the abundance of the proteins in the Acet supplementation condition:

(1) $$x-\mathrm{fold}\;\mathrm{change}=\frac{{\left(\mathrm{LFQ}\;\mathrm{of}\;\mathrm{Protein}\;Y\right)}_{\mathrm{Condition}\;A}}{{\left({\mathrm{LFQ}}_{Min}\;\mathrm{of}\;\mathrm{Protein}\;Y\right)}_{\mathrm{Acet}}}$$

If the average LFQ value of the Acet cultivation is zero, the smallest LFQ values becomes the reference. Heat maps were created by conditional formatting in Excel 2016 (Microsoft Corporation, Redmond, Washington, DC, USA) and the multi-layer growth curve in was designed using JMP Pro 15 (SAS Institute GmbH, Heidelberg, Germany).

## 3. Results

### 3.1. Cultivation of P. bryantii B_1_4 Using Single SCFAs

Growth of *P. bryantii* B_1_4 in M2-A medium was observed if at least one SCFA was added to the medium. Without addition of SCFAs, no growth was detected within the monitored period of 24 h. Applied SCFAs were acetic (Acet), propionic (Prop), butyric (But), iso-butyric (iBut), valeric (Val) and isovaleric acid (iVal) at a concentration of 15 mM. The maximal optical density (OD) in the medium M2-A after 8 and 24 h respectively, was observed for the cultivation with Val (OD_8h_ = 1.73; OD_24h_ = 1.77). In contrast, iVal supplementation exhibited the lowest cell density (OD_8h_ = 1.16; OD_24h_ = 1.54; Table 1). The remaining cultivation conditions (Acet, Prop, But, and iBut) showed different values for the 8 h and the 24 h incubation. Trends of higher cell densities were found in the Val and Acet cultures, while iVal supplementation revealed a lower OD. Replicates of the presented average of the optical density in Table 1 can be seen in Table S3. The relative changes in pH between the beginning and end of the incubation is indicated by the acidification (∆pH) in Table 1 and can be seen in detail in Table S3. Highest acidifications were found in cultures supplemented with Val (∆pH_8h_ = 1.47; ∆pH_24h_ = 1.62), followed by But (∆pH_8h_ = 1.33; ∆pH_24h_ = 1.61). The lowest acidification was also found in cultures with iVal (∆pH_8h_ = 0.56; ∆pH_24h_ = 1.05). The consumption or production of the single SCFAs was calculated by subtracting the concentration in the non-inoculated media from the final concentration in the cultures. The fermentation products of *P. bryantii* B_1_4 should be formic, succinic, lactic, and acetic acid [31,32]. Changes in SCFA concentrations are considered as trends, except for acetic acid, which showed a larger variation from 6–12 mM in 8 h and from 11–17 mM in 24 h. Consumption of SCFAs was about 0.5 mM within 8 h and 1.0 mM within 24 h for the other SCFAs. Small amounts of isovaleric acid were found in all cultures, except iVal. In addition, *P. bryantii* B_1_4 exhibited cell sedimentation when isovaleric acid was supplemented.

Other carboxylic acids, such as lactic, succinic, and formic acid, did not promote growth of *P. bryantii* B_1_4 as the used SCFAs did, which narrows the structural properties of the required acid sources. Growth promoting compounds most likely consist of one carboxyl group and an alkyl attachment of at least one methyl group (CH_3_-[CH_2_]_n_-COOH; *n* = 0–3).

### 3.2. Proteome Inventory of P. bryantii B_1_4

Bacterial cells were harvested from M2-A media at the end of the exponential phase. The overall cultivation characteristics for the proteome approach are given in the supplemental data (Figure S1). The number of quantified proteins per sample ranged from 1518 to 1599 (Table S4). In total, 1818 proteins were detected. The proteome shared by all cultures comprised 1437 proteins with an LFQ-value above zero in at least two out of three replicates. This number covered 38% of all predicted proteins or gene products (3776 proteins) listed in the UniProt database for *P. bryantii* B_1_4 (2 July 2019). Out of all identified proteins, 1230 proteins could be assigned to a KEGG orthology (KO) number (68%) and 1734 proteins to a cluster of orthologous groups (COG) classification (95%).

**Figure 1:**
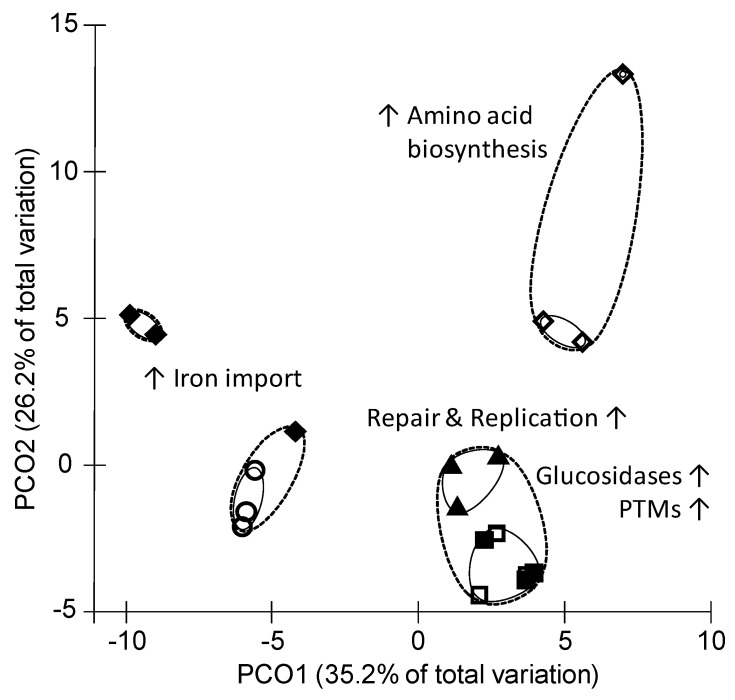
Assignment and comparison of pathways and cellular functions in *P. bryantii* B_1_4 exposed to various short-chain fatty acids based on relative protein abundances. Principle component analysis was done based on S17 Bray Curtis similarity out of the resemblance matrix. Elevated main protein functions causing the separation of the different proteomes are indicated close to the clusters. Clusters illustrate a similarity of either 88% (dotted line) or 92% (solid line). Supplementations with single SCFAs are displayed by following symbols: Acet (○), Prop (▲), But (◻), iBut (■), iVal (◇), Val (◆). PTM: post translational modifications. The clustering of supplementation conditions was significant by a *p*-value < 0.05 (Monte Carlo correction).

Comparisons of protein abundances of all samples revealed 84% similarity between all cultures. Cultures clustered in the principle component (PCO) plot mostly regarding their supplementations (*p* < 0.03; Figure 1). A grouping toward the SCFA property (chain length, iso-formation) was not observed. Clustering of cultures according to proteome analysis was accompanied with a change in abundance of several proteins representing specific functional groups (Figure 1). Figures S2 and S3 provide information about most and least abundant COG protein groups within the SCFA supplementations. Note that not all identified proteins could be assigned into COG class or labeled with a KO number. Upon cells cultivated with Val, the proteome contained numerous highly abundant proteins belonging to ribosome and porphyrin metabolism (Figure S4), while iVal cultures revealed most abundant proteins of the amino acid biosynthesis (Figure S5). Val and iVal cultures together showed more abundant proteins for nucleotide metabolism (Figure S2). Similar to the Val supplementation, the Prop cultures led to an increased abundance of ribosomal proteins as well as replication and repair proteins (Figure S6). Heat shock proteins (HSP) and chaperonin increased slightly in the Val, Prop, and iVal cultures (Figure S2). Many outer membrane proteins were elevated in Acet (Figure S7) especially iron import related proteins were elevated together with Val (Figure S8). Cultures with butyric acid and isobutyric acid had a minor number of culture specific proteins including glucosidases and proteins for post translational modification. 

**Figure 2:**
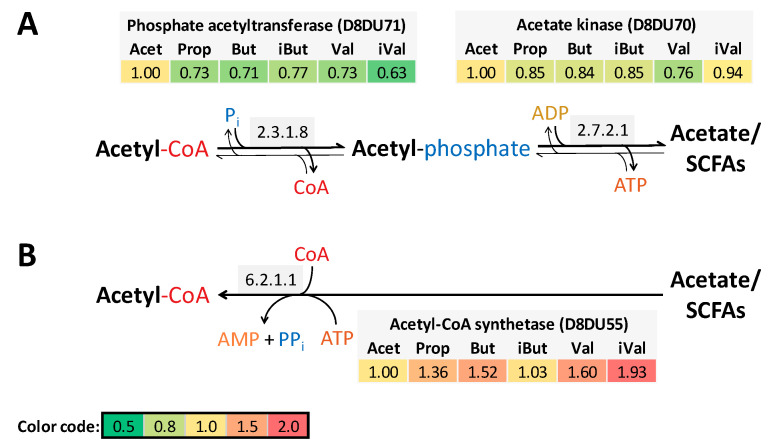
Routes of acetate formation and acetate utilization in response to SCFA exposure of *P. bryantii* B_1_4. The abundance of the mentioned enzymes (Uniprot ID) is represented by relative abundance (*n* = 3) for every treatment. Protein abundance of Acet cultivation is set to one. Above the reaction arrows are the EC numbers of the enzymes displayed. The color code is given in the lower left corner. (**A**) Acetyl-phosphate path with bidirectional reactions, where production of acetate is more likely due to ATP formation. (**B**) Reaction via the acetyl-CoA synthetase for SCFA assimilation, explicitly seen for iVal.

Two pathways for acetate utilization were detected in *P. bryantii* (Figure 2). Acetate is most likely formed via the acetate kinase and phosphate acetyltransferase (Figure 2A). Supplementation with acetic acid showed the greatest enzyme elevation in the pathway via acetyl-phosphate. The single enzyme reaction via the acetyl-CoA synthetase with ATP and coenzyme A was elevated (1.3–1.9-fold) in most of cultures except of iBut and Acet (Figure 2B). Enzymes of the two-step reaction (Figure 2A) were in total 22–65-times more abundant for acetate kinase and 3–13-times more abundant in phosphate acetyltransferase as in comparison the acetyl-CoA synthetase, based on the LFQ-values obtained from MaxQuant. 

**Figure 3:**
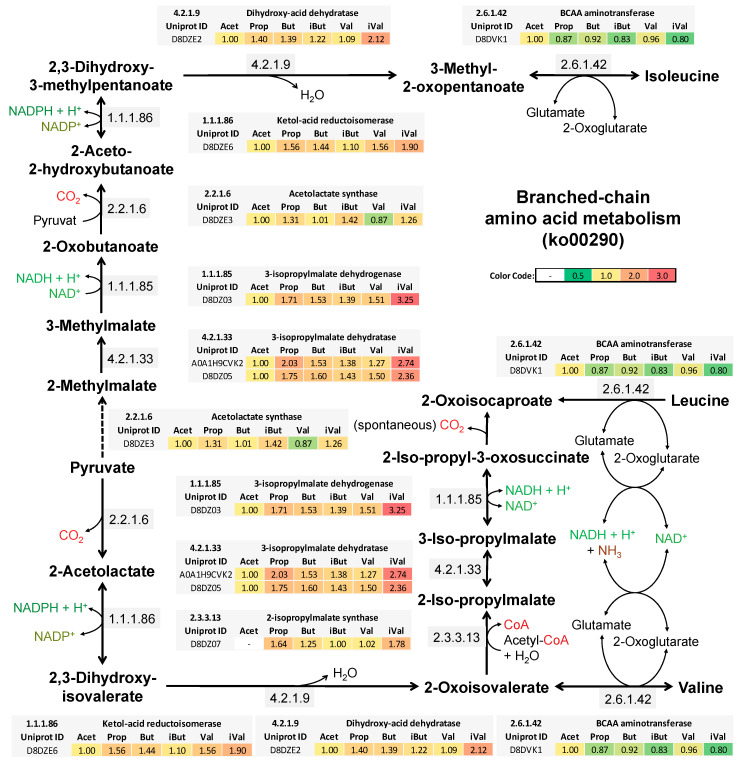
Influence of SCFAs on the relative abundance of enzymes required for the conversion of pyruvate to branched-chain amino acids in *P. bryantii* B_1_4. This pathway is based on the KEGG pathway (ko00290). Grey boxes indicate the mean relative abundance (*n* = 3) of the enzymes (including EC numbers close to the arrows) and their detected subunits or copies, as shown by their Uniprot IDs. The color code on the right side displays fold change with respect to the Acet condition, which is set to one. Dotted arrow illustrates an undetected enzyme.

Figure 3 displays the synthesis pathway for the branched-chain amino acids (BCAAs) valine, leucine, and isoleucine including enzymatic cofactors, the EC number, and the enzyme abundance for the respective cultures. An elevation of the BCAA synthesis enzymes was found especially in the iVal cultures within the reaction path from pyruvate to the final intermediate of the BCAA biosynthesis. Required enzymes for BCAA synthesis were a set of seven enzymes of which six were found in branches for valine, leucine, and isoleucine synthesis. Most of those enzymes were at least two-fold elevated in iVal, except for the acetolactate synthase (EC 2.2.1.6) and the BCAA aminotransferase (EC 2.6.1.42). 

The similarity percentage (SIMPER) analysis of the total dataset included mostly proteins with high overall abundance and fold-change (Figure S6). The majority of proteins identified by the SIMPER analysis belonged to the central carbon metabolism (enolase, fructose-1,6-bisphosphate aldolase, glycerin-aldehyde-3-phosphate dehydrogenase, L-fucose isomerase, pyruvate phosphate dikinase, etc.) and were mainly increased in iVal cultures. Proteins related to translation and transcription were elevated in iVal too. Certain iron related proteins, including imelysin, were more abundant in the Acet and Val cultures (Figure S6). The iBut supplementation led also to an elevation of some outer membrane proteins and two tetratricopeptide (TRP) domain proteins, which were also more abundant in iVal. 

Proteins related to iron import were found in high abundances in *P. bryantii* B_1_4 cultures Acet and some less in Val (Figure S8). This includes TonB transporters, iron utilization proteins and transcription factors. The heme and ferrienterochelin import is mostly conducted by TonB associated proteins, like HmuY or the ferrienterochelin receptor. The HmuY (D8E002) was found up to 8-times more abundant in Acet and Val when compared to the iVal cultivation condition. In the residual conditions, HmuY was around five-fold less abundant when compared to Acet. The outer membrane receptor for ferrienterochelin (D8E003) showed a 10-fold increase in Acet cultures and eight-fold in Val cultures while being low abundant (1–2-fold) in the remaining cultures (reference one-fold in iVal). Multiple copies of TonB proteins and uncharacterized proteins with homology to iron transporters were detected (Figure S8). The iron acquisition protein hemolysin (D8DU89) was found in a two-fold amount in the iVal and 1.5-fold in Val while remaining cultures exhibited around one-fold magnitudes. 

### 3.3. Effect of SCFA Exposure on Long-Chain Fatty Acids in Membranes from P. bryantii

**Figure 4:**
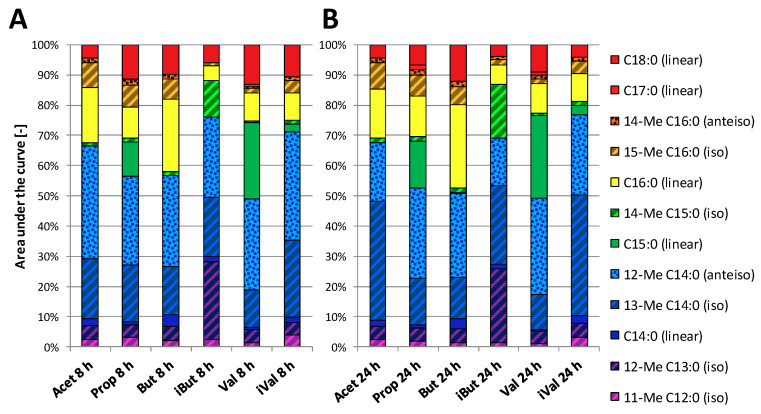
Influence of SCFAs on long-chain fatty acid (LCFA) profiles in *P. bryantii* B_1_4 cell membranes. (**A**) LCFA profiles after 8 h (*n* = 1) and (**B**) after 24 h of incubation (*n* = 1) are presented as relative abundances of LCFA using AUC values. The nomenclature of the LCFA is defined by the maximal chain length and number of carbon atoms, given by the capital C, followed by the number of carbon double bonds after the colon. The position of methylation (Me) is described by the number in front while the type of branching is given in brackets and was implemented with respective patterns. Linear LCFAs (without pattern), iso-branched (diagonal upward stripes), anteiso-branched (dotted pattern).

In order to investigate SCFA incorporation into long-chain fatty acids (LCFAs), the fatty acids of freeze-dried cells from *P. bryantii* B_1_4 were quantified by GC-MS. The analyses showed that an increase of certain iso- and anteiso- LCFA was concomitant to the supplemented iso- and anteiso-SCFA (Figures S9 and S10 and Tables S5 and S6). The iBut supplementation resulted in higher amounts of 12-methyl C13:0 and 14-methyl C15:0 (Figure 4). Both LCFAs were odd-chained and iso-branched like the supplemented isobutyric acid. Compared to the other conditions, iVal showed a small increase of the iso-branched 11-methyl C12:0 after 24 h of incubation. Additionally, a minor amount of the linear C15:0 was measured in iVal, but the major amounts of this odd chained acid were found in Val and Prop. Larger quantities of C16:0 and 15-me C16:0 were found in But, Prop, and Acet. Minor amounts of C17:0 were recovered in Prop and Val. The fatty acid 12-methyl- and 13-methyl C14:0 were the predominant LCFAs in all cultivation conditions, whereby the predominance of 12-methyl C14:0 shifted in some conditions to 13-methyl C14:0 during incubation from 8–24 h. The consumption of the supplemented SCFAs was between 0.2–0.5 mM for 8 h and reached a maximal depletion of 1.0 mM after 24 h of incubation (Table 1). Furthermore, SCFA measurements tend to confirm a minor SCFA consumption in cultivations for LCFA investigations (Table S7) as well as for the proteome investigation (Table S8).

### 3.4. Effect of Carbon Source on Growth of P. bryantii 

The results described above corroborate the suitability of the M2-A medium to cultivate *P. bryantii* B_1_4 and demonstrate that SCFAs are incorporated in lipid membrane synthesis. Still, the major energy supporting carbon source for *P. bryantii* B_1_4 are sugars derived from polysaccharides like hemicellulose or pectin. It was tested if glucose is usable as a sole energy supporting carbon source by omitting maltose, cellobiose and lactate from the medium (M2-B). Growth kinetics showed a lag-phase of about 120 min and an increase in OD of about 0.9 per hour during the exponential phase. 

**Figure 5:**
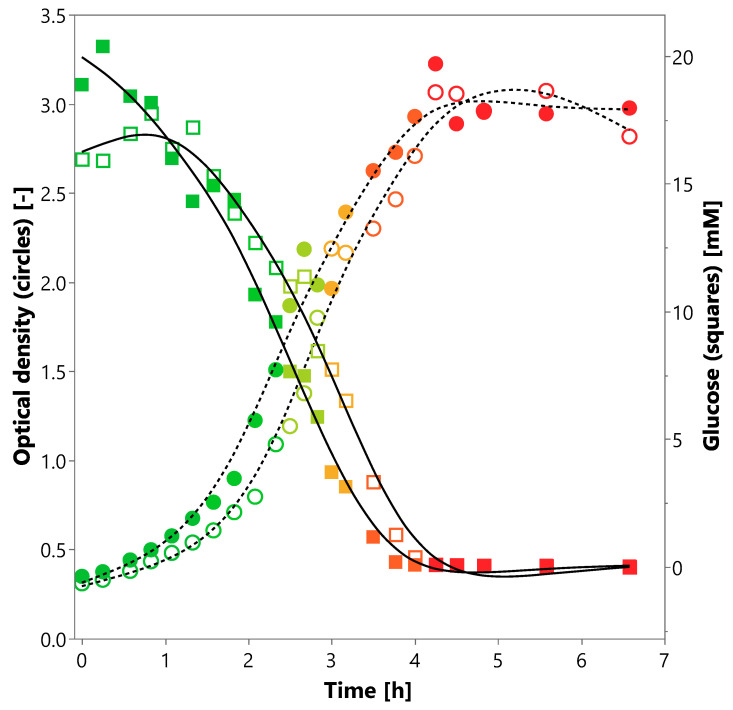
Growth of *P. bryantii* B_1_4 in minimal medium (M2-B) supplemented with glucose as the only energy supporting carbon source. Following parameters are given for a duplicate (A: empty, B: filled symbols) of a two-liter batch culture: optical density at 600 nm (circles), glucose concentration in millimolar (squares) and pH as a color gradient from pH 7 (green) to pH 5.2 (red). The interpolation curves for optical density (dotted line) and glucose (solid line) represent the trend for each duplicate.

Maximum cell densities were observed after 4.25 h using 10% (*v*/*v*) inoculation volume. The pH dropped about 1.5 and stopped decreasing simultaneously, when glucose was almost depleted at 220 min (Figure 5). A cell yield of 0.39 g cells per gram glucose was achieved in the present batch culture. Growth and fermentation activity occurred in the presence of glucose emphasizing the dispensability of maltose or cellobiose (Figure 5). All raw data are listed in the appendix (Table S9). Additional cultivations with a mixture of SCFAs, but without sugars as carbon sources showed no growth within 24 h (data not shown).

## 4. Discussion

### 4.1. SCFAs Replace Rumen Fluid for Cultivation of P. bryantii B_1_4

This study verified that rumen fluid can be replaced by various SCFAs to cultivate *P. bryantii* B_1_4. iVal cultures showed the slowest growth indicated by lower optical densities and a tendency of cells to aggregate (Figure S11), which are both indicators of stress [33]. In accordance, cells exposed to iVal showed increased abundance of stress-related chaperones. Absence of any SCFAs resulted in growth of *P. bryantii* B_1_4 only after 36 h of incubation. A further possibility is the selection and reproduction of a sub-population of *P. bryantii* cells, which are less dependent on SCFA. The first culture inoculation allowed a transfer of sufficient amounts of SCFAs from M2-A medium as cells were not washed resulting in a short lag-phase of 3 h (Figure S12). The second transfer in SCFA-free media resulted in a prolonged lag-phase of at least 6 h. Third transfer of *P. bryantii* B_1_4 in SCFA-free media showed the adapted subpopulation as lag-phase was again 3 h (Figure S12). This points towards SCFAs not being essential in the central energy-producing carbon metabolism but enhance growth of *P. bryantii* B_1_4. Figure 2A showed a part of the fermentative branch to generate ATP by producing acetate, a more common reaction. Furthermore, acetate kinase has been described to be responsible for excretion of excessive carbohydrates in form of acetate and is therefore a catabolic repressed enzyme [34,35]. Despite acetic acid supplementation, further acetate was produced presumably via the acetyl-phosphate pathway, meaning that catabolite repression was not activated under culture conditions of the present study. Assimilation of SCFA via acetyl-CoA synthetase activity was reduced in Acet and iBut compared to Prop, But, Val and iVal supplementations. A catabolite repression especially for Acet is proposed as discussed by Valgepea, K., et al. [35]. Although the reported cultivation conditions are artificial and do not represent the natural environment of *P. bryantii*, they may provide a deeper understanding of SCFA requirement, anabolism and related broad range of effects. 

### 4.2. SCFA Influence the Abundance of Iron Transport Proteins 

*P. bryantii* is a close relative to pathogenic *Prevotella* species such as *P. denticola* and *P. bivia* that depend on the acquisition of iron, which is important for survival within the host [36]. Enteroferrin, HmuY, TonB, and other outer membrane proteins were elevated in *P. bryantii* cultures containing Acet and iVal. This suggests a regulation of the iron import by supplementation of certain fatty acids or their derivatives. *P. intermedia* 17 showed an increase of a HmuY-like protein and a TonB-linked outer membrane receptor when iron sources were restricted [37]. Iron deficiency in ruminants is rarely observed in adult ruminants and should not influence growth of *Prevotella* species in the rumen at proper feeding conditions [38]. In the study of Herold, S., et al. [39], an increase of TonB was found in a shiga-toxigenic *E. coli* when multiple SCFAs were supplemented. Another heme importing protein is hemolysin, which was increased in iVal and is usually also expressed under iron deficiency. Iron can be imported in connection to porphyrin or chelating agents such as citrate or enteroferrin. For some *Prevotella* sp., it was demonstrated that heme (an Fe^3+^ bound porphyrin) has to be present in a concentration of at least 10 nM in order to obtain sufficient growth [40]. Investigating the porphyrin biosynthesis pathway revealed enzymes, which convert L-glutamate into protoporphyrinogen IX (PPIX) and coproporphyrinogen. However, the final enzyme for producing porphyrin is not annotated for *P. bryantii* B_1_4 or other *Prevotella* species (Figure S13). The absence of the final enzyme may not be relevant since a PPIX supplementation for *P. intermedia* resulted in sufficient growth [41]. Nevertheless, the presence of functional porphyrin metabolism was not shown in the proteomic dataset. Thus, the role of heme and its supplementation remains uncertain. Possible heme utilization as an oxygen protectant layer on the cell surface was found in *P. nigerescens* and *P. intermedia* [42,43]. Furthermore, heme can contribute to the performance of cytochrome containing enzymes and the electron transport chains as *P. bryantii* B_1_4 uses anaerobic respiration [44,45]. In the study from Sperry, J., et al. [46], a depletion of heme resulted in an increase of lactate and fumarate, which was not confirmed by the current study. Until now, very little is known about the regulation of the porphyrin biosynthesis in non-photosynthetic bacteria and catabolic repression by heme was already reported for other bacterial species [47]. A higher request for the porphyrin ring instead of iron is indicated by the current findings as well as by the study of Caldwell, D., et al. [40]. It can be assumed that acetic and valeric acid increase the abundance of heme import proteins in *P. bryantii*, which in turn enhance heme uptake and regulate the porphyrin biosynthesis as also described by Granick and Beale [47]. An enhanced heme uptake due to the direct aid of SCFAs can be neglected since heme is a large molecule and soluble in alkaline solutions. Absence of mineral iron in the media would have also caused similar patterns among all cultivation conditions and can therefore be also excluded as a trigger. An iron-deficiency was not seen in bacterial density and growth behavior in *P. bryantii* in rumen fluid containing medium (M2) versus newly created growth medium (M2-A) with hemin.

### 4.3. SCFAs are Incorporated into Long-Chain Fatty Acids of Lipid Membranes 

Membranes are composed of LCFAs, which require a malonyl-CoA with an acyl-CoA or acyl-carrier-protein as a primer molecule. This primer molecule is assumed to be a SCFA. The hypothesis is verified by rediscovering features of SCFA in LCFAs. The analyses of LCFA in the current study were similar to LCFA ratios of other *Prevotella* sp. in regard to the predominant fatty acids 13-me C14:0 and 12-me C14:0 [48,49]. The beneficial effects of incorporating branched SCFAs into the membrane of *P. bryantii* B_1_4 can mirror an adaptation to antibacterial peptides, low temperature and low pH [50,51,52,53,54]. The latter adaptation is expected for the LCFAs 11-me C12:0 (iso) and 13-me C14:0 (iso). Both LCFAs correlated with the pH at 8 h and 24 h with a correlation coefficient between R = −0.74 and −0.90, indicating a possible connection between LCFAs and acidity. Referring to SCFA implementation into LCFAs, acetic acid seems to be the universal building block for the requirements of *P. bryantii* B_1_4 to form all necessary LCFAs with a large fraction of even numbered and branched LCFAs. Other SCFAs shift the LCFA profile regarding their traits (branching, chain length). While traits of SCFAs were recovered for most of the cultures, iVal lacked in larger LCFA with such traits, meaning that isovaleric acid is preferably used for other synthesis pathways like BCAA syntheses. Other rumen bacteria such as *Ruminococci* showed incorporation of larger iso- and odd-chain LCFA [21]. The uptake mechanisms of SCFA to be available in the cell is only known for a few microorganisms and shortly explained in Reiser, K., et al. [55] and DiRusso, C.C. and Black, P.N. [56]. In the present dataset, fatty acid transport protein FadL (D8DTK4) showed a decreasing abundance with higher chain length (R = −0.89), which may explain the protein elevation for fatty acid related proteins in Acet. Besides FadL, LCFA-transport proteins (A0A1H9JWM8, D8DZW6) are suspected to be also capable of carrying SCFA through the membrane [56]. The two quantified LCFA-transport proteins were twice as much (A0A1H9JWM8) or similarly (D8DZW6) abundant in iVal as in Acet but almost not affected by other SCFAs. Findings on growth, proteome and SCFA assimilation together indicate a bioactive role for isovaleric acid in *P. bryantii* B_1_4 cells. 

### 4.4. Isovaleric Acid Enhance Branched-Chain Amino Acid Synthesis

Data of SCFA measurements showed low amounts isovaleric acid in all culture conditions, except for iVal, which is most likely connected to the degradation of tryptic digested casein or other high protein substrates [57,58]. Thus, *P. bryantii* B_1_4 seems to be capable of producing low amounts of branched SCFA under supplementation of a protein source. Enzymes involved in the BCAA metabolism became elevated in iVal cultures, while other SCFA supplements showed only low abundances of these proteins. Furthermore, a transformation from isovaleric acid to BCAAs is assumed by a lower specificity of the acetyl-CoA synthetase as shown in Glasemacher, J., et al. [59]. Especially the 3-isopropylmalate dehydrogenase (3-IPM DH) with an approximate three-fold elevation was highlighting the BCAA metabolism for the iVal condition. All co-elevated enzymes of iVal in the amino acid and carbon metabolism may react to enhanced concentration of isovaleric acid derived compounds, like on BCAAs, to avoid intoxication [60]. The study of Allison, M.J., et al. [61] observed a higher activity of the IPM synthase under presence of isovaleric acid plus valine. In contrast, isopropylmalate synthase was inhibited under leucine supplementation in *Bacteroides* species, suggesting a substrate regulation via the CodY operon [62]. The CodY operon seem to react to BCAA and GTP concentration, but was not identified for *P. bryantii* B_1_4 [63]. Atasoglu, C., et al. [64] discovered that nitrogen content derived less from ammonia but more from peptides when trypticase supplementation was increased from 1–10 g/L. This means that *P. bryantii* B_1_4 might have shown stronger effects of amino acid synthesis when less trypticase is supplemented. BCAA pathway showed the most prominent elevations of related proteins among all other amino acid synthesis pathways. This reveals a direct impact of isovaleric acid on their abundance. Only the BCAA aminotransferase was low in abundance, which was probably connected to the diminished ammonia assimilation [64].

## 5. Conclusions

Driven by exploring the reason for SCFA requirement of *Prevotella bryantii* B_1_4 insights about single SCFA supplementation revealed different outcomes with respect to growth performance and protein inventories. Parts of SCFAs were found in membrane lipids and amino acids. High bacterial cell densities were measured for Val, while OD measurements at 24 h were lowest for the iVal condition. Cell sedimentation in the supplementation with iVal indicated a challenge for *P. bryantii*, which was most likely be caused by the overload of isovaleric acid and peptides from fermentation of tryptic digested casein. The SCFA measurements verified acetic acid production and consumption of supplemented SCFAs. Furthermore, incorporation of SCFA was confirmed by recovering traits in LCFAs of the lipid membrane. Most proteomes of single SCFA cultures shared 80% of their quantified proteome profile. Differences occurred in proteins responsible for carbon metabolism, porphyrin metabolism (Val), outer membrane proteins, especially porphyrin import related proteins (Acet, Val) and amino acid biosynthesis (iVal). Latter demonstrated the regulation of the BCAA synthesis by isovaleric acid. Nevertheless, proteome analysis of *P. bryantii* B_1_4 cells cultivated with single SCFAs narrowed the range of potential mechanisms and pinpointed contributing of SCFA to growth and other possible functions. 

## Figures and Tables

**Table 1:** Effect of short-chain fatty acids on growth, acidification, and short-chain fatty acid (SCFA) conversion in *Prevotella bryantii* B_1_4. *P. bryantii* was grown in M2-A medium supplemented with the indicated SCFA. Two sets of experiments were performed, each with three cultures obtained by consecutive transfer of inoculum. In the first set, each of the three cultures was analyzed for optical density (OD), pH, and SCFA composition after 8 h. In the second experiments, these parameters were determined after 24 h. The values for mean and standard deviation (SD) of one set (*n* = 3) are reported in Table S3. The acidification of the medium (∆pH) is given as a relative value for each cultivation condition. The consumption (negative value) or production (positive value) of acetic acid (C2), propionic acid (C3), butyric acid (C4), isobutyric acid (iC4), valeric acid (C5), and isovaleric acid (iC5) are displayed in millimolar (*n* = 1). Expect for acetic acid (C2), the observed variations in SCFA concentrations were in the range of ±1.5 mM and therefore were not considered to be significant.

Cultivation Condition		Mean ± SD of OD_600_		∆pH	Production/Consumption (mM) of ____					
					C2	C3	iC4	C4	iC5	C5
**8 h incubation**	Acet	1.44	±0.19	1.34	9.55	0.00	0.00	0.00	0.06	0.00
	Prop	1.50	±0.17	1.32	10.03	0.70	0.00	−0.04	0.08	0.07
	But	1.43	±0.16	1.33	10.14	−0.05	0.00	−0.10	0.09	0.00
	iBut	1.23	±0.07	0.85	6.06	0.00	−0.49	0.00	0.08	0.00
	Val	1.73	±0.04	1.47	11.77	0.00	0.00	−0.03	0.16	−0.69
	iVal	1.16	±0.09	0.56	6.21	0.00	0.00	0.00	−0.24	0.00
**Cultivation** **Condition**		**Mean ± SD of OD_600_**		**∆pH**	**Production/Consumption (mM) of ____**					
					**C2**	**C3**	**iC4**	**C4**	**iC5**	**C5**
**24 h incubation**	Acet	1.67	±0.04	1.38	13.94	0.00	0.00	0.00	0.08	0.00
	Prop	1.64	±0.26	1.57	16.05	0.27	0.00	−0.04	0.07	0.06
	But	1.66	±0.15	1.61	16.79	−0.10	0.00	−0.43	0.10	0.00
	iBut	1.64	±0.06	1.49	14.02	0.00	−0.87	0.00	0.09	0.00
	Val	1.77	±0.13	1.62	15.59	0.00	0.00	−0.01	0.17	−0.89
	iVal	1.54	±0.34	1.05	10.82	0.00	0.00	0.00	−0.46	0.00

## Supplementary Materials

The following are available online at https://www.mdpi.com/2227-7382/8/4/28/s1, Table S1. Composition of media for growth of *P. bryantii* B_1_4.; Table S2. Vitamin Solution for *P. bryantii* B_1_4 cultivation media M2-A and –B.; Table S3. Optical density and pH of cultivations for LCFA harvesting. Table S4. Numbers of peptides and proteins determined by LC-MS/MS measurements. Table S5. Cultivation parameters and relative amounts of trait specific long chain fatty acids. Table S6. Percentile amount of long-chain fatty acids found in lipid membrane of *P. bryantii* B_1_4. Table S7. SCFA measurement of cultivation for LCFA analysis. Table S8. SCFA measurement from supernatant of cultivation for proteomic analysis. Table S9. Growth parameters of *P. bryantii* B_1_4 in M2-B. Figure S1. Cultivation of *P. bryantii* B_1_4 in M2-A medium in the presence of various short-chain fatty acids for subsequent proteome analyses. Figure S2. Count of maximal abundant COGs per SCFA supplementation. Proteins which were maximal expressed among all cultures were counted and grouped by their functionality using the cluster of orthologous groups (COGs). Figure S3. Count of least abundant COGs per culture conditions. Proteins which were least expressed among all cultivation conditions were counted and grouped by their functionality using the cluster of orthologous groups (COGs). Figure S4. Porphyrin synthesis pathway in *P. bryantii* B_1_4 with enzyme abundances. Figure S5. Heat map of enzymes from the amino acid synthesis pathway (ko01230). Figure S6. Similar percentage (SIMPER) analyzed proteins with >1% contribution. Figure S7. Heat map of most abundant outer membrane proteins. Figure S8. Acet and Val culture specific proteins. Figure S9. Percentile distribution of long-chain fatty acids (LCFA) by traits of chain length and branching structure after 8 h incubation. Figure S10. Percentile distribution of long-chain fatty acids (LCFA) by traits of chain length and branching structure after 24 h incubation. Figure S11. iVal cultivation of *P. bryantii* B_1_4 in Hungate tubes Figure S12. Growth curve of cultures transferred into SCFA-free media. Media composition was similar except the addition of any SCFAs Figure S13. KEGG pathway of porphyrin and chlorophyll metabolism found in all cultivation conditions.

## References

[1] Flint H.J., Bayer E.A. (2008). Plant cell wall breakdown by anaerobic microorganisms from the mammalian digestive tract. Ann. N. Y. Acad. Sci..

[2] Dehority B. (1966). Characterization of several bovine rumen bacteria isolated with a xylan medium. J. Bacteriol..

[3] Duncan S.H., Hold G.L., Harmsen H.J., Stewart C.S., Flint H.J. (2002). Growth requirements and fermentation products of Fusobacterium prausnitzii, and a proposal to reclassify it as Faecalibacterium prausnitzii gen. nov., comb. nov. Int. J. Syst. Evol. Microbiol..

[4] Dehority B., Scott H., Kowaluk P. (1967). Volatile fatty acid requirements of cellulolytic rumen bacteria. J. Bacteriol..

[5] Torrungruang K., Jitpakdeebordin S., Charatkulangkun O., Gleebbua Y. (2015). Porphyromonas gingivalis, Aggregatibacter actinomycetemcomitans, and Treponema denticola/Prevotella intermedia co-infection are associated with severe periodontitis in a thai population. PLoS ONE.

[6] Tompkins G.R., Wood D.P., Birchmeier K.R. (1997). Detection and comparison of specific hemin binding by Porphyromonas gingivalis and Prevotella intermedia. J. Bacteriol..

[7] Scott H., Dehority B. (1965). Vitamin requirements of several cellulolytic rumen bacteria. J. Bacteriol..

[8] Gibbons R.J., Macdonald J.B. (1960). Hemin and vitamin k compounds as required factors for the cultivation of certain strains of Bacteroides melaninogenicus. J. Bacteriol..

[9] Alvarez H., Santini F.J., Rearte D.H., Elizalde J.C. (2001). Milk production and ruminal digestion in lactating dairy cows grazing temperate pastures and supplemented with dry cracked corn or high moisture corn. Anim. Feed Sci. Technol..

[10] Byrne C., Chambers E., Morrison D., Frost G. (2015). The role of short chain fatty acids in appetite regulation and energy homeostasis. Int. J. Obes..

[11] Von Engelhardt W., Rönnau K., Rechkemmer G., Sakata T. (1989). Absorption of short-chain fatty acids and their role in the hindgut of monogastric animals. Anim. Feed Sci. Technol..

[12] LeBlanc J.G., Chain F., Martín R., Bermúdez-Humarán L.G., Courau S., Langella P. (2017). Beneficial effects on host energy metabolism of short-chain fatty acids and vitamins produced by commensal and probiotic bacteria. Microb. Cell Fact..

[13] Fujita Y., Matsuoka H., Hirooka K. (2007). Regulation of fatty acid metabolism in bacteria. Mol. Microbiol..

[14] Takahashi N., Yamada T. (2000). Pathways for amino acid metabolism by Prevotella intermedia and Prevotella nigrescens. Oral Microbiol. Immunol..

[15] Bhatia S.K., Yang Y.-H. (2017). Microbial production of volatile fatty acids: Current status and future perspectives. Rev. Environ. Sci. Bio/Technol..

[16] Hobson P.N., Norris J.R., Ribbons D.W. (1969). Rumen bacteria. Methods in Microbiology.

[17] Cotta M.A., Russell J.B. (1982). Effect of peptides and amino acids on efficiency of rumen bacterial protein synthesis in continuous culture. J. Dairy Sci..

[18] Wischer G., Boguhn J., Steingaß H., Schollenberger M., Hartung K., Rodehutscord M. (2013). Effect of monensin on in vitro fermentation of silages and microbial protein synthesis. Arch. Anim. Nutr..

[19] Paczia N., Nilgen A., Lehmann T., Gätgens J., Wiechert W., Noack S. (2012). Extensive exometabolome analysis reveals extended overflow metabolism in various microorganisms. Microb. Cell Fact..

[20] Vlaeminck B., Fievez V., Cabrita A., Fonseca A., Dewhurst R. (2006). Factors affecting odd-and branched-chain fatty acids in milk: A review. Anim. Feed Sci. Technol..

[21] Allison M.J., Bryant M., Katz I., Keeney M. (1962). Metabolic function of branched-chain volatile fatty acids, growth factors for Ruminococci ii.: Biosynthesis of higher branched-chain fatty acids and aldehydes. J. Bacteriol..

[22] Deusch S., Camarinha-Silva A., Conrad J., Beifuss U., Rodehutscord M., Seifert J. (2017). A structural and functional elucidation of the rumen microbiome influenced by various diets and microenvironments. Front. Microbiol..

[23] Bradford M.M. (1976). A rapid and sensitive method for the quantitation of microgram quantities of protein utilizing the principle of protein-dye binding. Anal. Biochem..

[24] Jehmlich N., Schmidt F., Hartwich M., Bergen M.V., Richnow H.-H., Vogt C. (2008). Incorporation of carbon and nitrogen atoms into proteins measured by protein-based stable isotope probing (protein-sip). Rapid Commun. Mass Spectrom..

[25] Rappsilber J., Mann M., Ishihama Y. (2007). Protocol for micro-purification, enrichment, pre-fractionation and storage of peptides for proteomics using stagetips. Nat. Protoc..

[26] Olsen J.V., Godoy L.M.F.D., Li G., Macek B., Mortensen P., Pesch R., Makarov A., Lange O., Horning S., Mann M. (2005). Parts per million mass accuracy on an orbitrap mass spectrometer via lock mass injection into a C-trap. Mol. Cell. Proteom..

[27] Cox J., Hein M.Y., Luber C.A., Paron I., Nagaraj N., Mann M. (2014). Accurate proteome-wide label-free quantification by delayed normalization and maximal peptide ratio extraction, termed MaxLFQ. Mol. Cell. Proteom..

[28] Vizcaíno J.A., Csordas A., Del-Toro N., Dianes J.A., Griss J., Lavidas I., Mayer G., Perez-Riverol Y., Reisinger F., Ternent T. (2016). 2016 update of the pride database and its related tools. Nucleic Acids Res..

[29] Kanehisa M., Sato Y., Morishima K. (2016). BlastKOALA and GhostKOALA: KEGG tools for functional characterization of genome and metagenome sequences. J. Mol. Biol..

[30] Huerta-Cepas J., Forslund K., Coelho L.P., Szklarczyk D., Jensen L.J., von Mering C., Bork P. (2017). Fast genome-wide functional annotation through orthology assignment by EggNOG-Mapper. Mol. Biol. Evol..

[31] Hungate R.E. (1966). The Rumen and Its Microbes.

[32] Hackmann T.J., Ngugi D.K., Firkins J.L., Tao J. (2017). Genomes of rumen bacteria encode atypical pathways for fermenting hexoses to short-chain fatty acids. Environ. Microbiol..

[33] Conner R.L., Reilly A.E. (1975). The effects of isovalerate supplementation on growth and fatty acid composition of Tetrahymena pyriformis w. Biochim. Biophys. Acta.

[34] Grundy F.J., Waters D.A., Allen S., Henkin T. (1993). Regulation of the Bacillus subtilis acetate kinase gene by CcpA. J. Bacteriol..

[35] Valgepea K., Adamberg K., Nahku R., Lahtvee P.-J., Arike L., Vilu R. (2010). Systems biology approach reveals that overflow metabolism of acetate in Escherichia coli is triggered by carbon catabolite repression of acetyl-CoA synthetase. BMC Sys. Biol..

[36] Holt S.C., Ebersole J.L. (2005). Porphyromonas gingivalis, Treponema denticola, and Tannerella forsythia: The ‘red complex’, a prototype polybacterial pathogenic consortium in periodontitis. Periodontology 2000.

[37] Yu F., Anaya C., Lewis J.P. (2007). Outer membrane proteome of Prevotella intermedia 17: Identification of thioredoxin and iron-repressible hemin uptake loci. J. Proteom..

[38] Lindt F., Blum J.W. (1994). Occurrence of iron deficiency in growing cattle. Zent. Vet. A.

[39] Herold S., Paton J.C., Srimanote P., Paton A.W. (2009). Differential effects of short-chain fatty acids and iron on expression of iha in shiga-toxigenic Escherichia coli. Microbiology.

[40] Caldwell D., White D., Bryant M., Doetsch R. (1965). Specificity of the heme requirement for growth of Bacteroides ruminicola. J. Bacteriol..

[41] Leung K.-P., Folk S.P. (2002). Effects of porphyrins and inorganic iron on the growth of Prevotella intermedia. FEMS Microbiol. Lett..

[42] Smalley J.W., Silver J., Birss A.J., Withnall R., Titler P.J. (2003). The haem pigment of the oral anaerobes Prevotella nigrescens and Prevotella intermedia is composed of iron (iii) protoporphyrin ix in the monomeric form. Microbiology.

[43] Henry C.A., Judy M., Dyer B., Wagner M., Matthews J.L. (1995). Sensitivity of Porphyromonas and Prevotella species in liquid media to argon laser. Photochem. Photobiol..

[44] Macy J., Probst I., Gottschalk G. (1975). Evidence for cytochrome involvement in fumarate reduction and adenosine 5’-triphosphate synthesis by bacteroides fragilis grown in the presence of hemin. J. Bacteriol..

[45] Deusch S., Bok E., Schleicher L., Seifert J., Steuber J. (2019). Occurrence and function of the Na+-translocating NADH: Quinone oxidoreductase in Prevotella spp.. Microorganisms.

[46] Sperry J., Appleman M., Wilkins T.D. (1977). Requirement of heme for growth of Bacteroides fragilis. Appl. Environ. Microbiol..

[47] Granick S., Beale S.I. (1978). Hemes, chlorophylls, and related compounds: Biosynthesis and metabolic regulation. Adv. Enzymol. Relat. Areas Mol. Biol..

[48] Minato H., Ishibashi S., Hamaoka T. (1988). Cellular fatty acid and sugar composition of representative strains of rumen bacteria. J. Gen. Appl. Microbiol..

[49] Logar R.M., Zorec M., Kopečný J. (2001). Reliable identification of Prevotella and Butyrivibrio spp. From rumen by fatty acid methyl ester profiles. Folia Microbiol..

[50] Sun Y., Wilkinson B.J., Standiford T.J., Akinbi H.T., O’Riordan M.X. (2012). Fatty acids regulate stress resistance and virulence factor production for Listeria monocytogenes. J. Bacteriol..

[51] Mitchell N.J., Seaton P., Pokorny A. (2016). Branched phospholipids render lipid vesicles more susceptible to membrane-active peptides. Biochim. Biophys. Acta.

[52] Singh V.K., Hattangady D.S., Giotis E.S., Singh A.K., Chamberlain N.R., Stuart M.K., Wilkinson B.J. (2008). Insertional inactivation of branched-chain α-keto acid dehydrogenase in Staphylococcus aureus leads to decreased branched-chain membrane fatty acid content and increased susceptibility to certain stresses. Appl. Environ. Microbiol..

[53] Kaneda T. (1991). Iso-and anteiso-fatty acids in bacteria: Biosynthesis, function, and taxonomic significance. Microbiol. Rev..

[54] Sirobhushanam S. (2016). Alternative Pathway for Provision of Acyl CoA Precursors for Fatty Acid Biosynthesis: Purification and Kinetic Characterization of Phosphotransbutyrylase and Butyrate Kinase from Listeria Monocytogenes. Ph.D. Thesis.

[55] Reiser K., Davis M.A., Hynes M.J. (2010). Aspergillus nidulans contains six possible fatty acyl-CoA synthetases with FaaB being the major synthetase for fatty acid degradation. Arch. Microbiol..

[56] DiRusso C.C., Black P.N. (2004). Bacterial long chain fatty acid transport: Gateway to a fatty acid-responsive signaling system. J. Biol. Chem..

[57] El-Shazly K. (1952). Degradation of protein in the rumen of the sheep. 2. The action of rumen microorganisms on amino acids. Biochem. J..

[58] De Filippis F., Pasolli E., Tett A., Tarallo S., Naccarati A., De Angelis M., Neviani E., Cocolin L., Gobbetti M., Segata N. (2019). Distinct genetic and functional traits of human intestinal Prevotella copri strains are associated with different habitual diets. Cell Host Microbe.

[59] Glasemacher J., Bock A.K., Schmid R., Schönheit P. (1997). Purification and properties of acetyl-CoA synthetase (ADP-forming), an archaeal enzyme of acetate formation and ATP synthesis, from the hyperthermophile Pyrococcus furiosus. Eur. J. Biochem..

[60] Harris R.A., Joshi M., Jeoung N.H. (2004). Mechanisms responsible for regulation of branched-chain amino acid catabolism. Biochem. Biophys. Res. Commun..

[61] Allison M.J., Baetz A.L., Wiegel J. (1984). Alternative pathways for biosynthesis of leucine and other amino acids in Bacteroides ruminicola and Bacteroides fragilis. Appl. Environ. Microbiol..

[62] Commichau F.M., Forchhammer K., Stülke J. (2006). Regulatory links between carbon and nitrogen metabolism. Curr. Opin. Microbiol..

[63] Den Hengst C.D., Groeneveld M., Kuipers O.P., Kok J. (2006). Identification and functional characterization of the Lactococcus lactis CodY-regulated branched-chain amino acid permease BcaP (CtrA). J. Bacteriol..

[64] Atasoglu C., Valdés C., Walker N.D., Newbold C.J., Wallace R.J. (1998). De novo synthesis of amino acids by the ruminal bacteria Prevotella bryantii B14, Selenomonas ruminantium HD4, and Streptococcus bovis ES1. Appl. Environ. Microbiol..

