# Supplementary material for: Short-Chain Fatty Acids Modulate Metabolic Pathways and Membrane Lipids in Prevotella bryantii B14
Source: Proteomes. 2020 Oct 16;8(4):28. doi: 10.3390/proteomes8040028 (PMC7709123; doi:10.3390/proteomes8040028)
Supplement: Supplementary file 1 [file proteomes-08-00028-s001.pdf]

Supplementary Material

Short-Chain Fatty Acids Modulate Metabolic Pathways and Membrane Lipids in *Prevotella bryantii* B$_1$4

Andrej Trautmann [1], Lena Schleicher [2], Simon Deusch [1], Jochem Gätgens [3], Julia Steuber [2] and Jana Seifert [1,*]

[1] Institute of Animal Science, University of Hohenheim, 70599 Stuttgart, Germany; Andrej.Trautmann@uni-hohenheim.de (A.T.); deusch@uni-hohenheim.de (S.D.)

[2] Institute of Biology, University of Hohenheim, 70599 Stuttgart, Germany; lena.schleicher@uni-hohenheim.de (L.S.); julia.steuber@uni-hohenheim.de (J.S.)

[3] Institute of Bio- and Geosciences, IBG-1: Biotechnology, Forschungszentrum Jülich, 52425 Jülich, Germany; j.gaetgens@fz-juelich.de

* Correspondence: jseifert@uni-hohenheim.de

Table S1. Vitamin Solution for *Prevotella bryantii* B$_1$4

Vitamin solution was added after the autoclaving to achieve the final concentration in the media M2-A and –B.

Ingredients	Molecular weight [g/mol]	Weight / Volume		Final concentration in culture	
Hemin	651.94	1.00	mg/L	1,534	nM
Menadion	172.18	0.60	mg/L	3,485	nM
Folic acid	441.40	0.05	mg/L	113	nM
Thiamine hydrochloride	337.27	2.00	mg/L	5,930	nM
Riboflavin	376.37	2.00	mg/L	5,314	nM
Nicotinamide	112.12	2.00	mg/L	17,838	nM
Pyridoxamine dihydrochloride	241.11	2.00	mg/L	8,295	nM
Ca-Pantothenate	476.54	2.00	mg/L	4,197	nM
Aminobenzoic acid	137.14	0.10	mg/L	729	nM
Biotin	244.31	0.05	mg/L	205	nM
Cyanocobalamin	1579.60	0.05	mg/L	32	nM

Table S2. Composition of media for growth of *P. bryantii* B_14.

Ingredients	Weight/Volume		Final concentration in culture	
$NaHCO_3$	4.00	g/L	47.62	mM
NaCl	0.90	g/L	15.40	mM
$(NH_4)_2SO_4$	0.90	g/L	6.81	mM
KH_2PO_4	0.45	g/L	3.31	mM
$K_2HPO_4 \times 3 H_2O$	0.45	g/L	1.97	mM
$MgSO_4 \times 7 H_2O$	0.10	g/L	0.61	mM
$CaCl_2 \times 2 H_2O$	0.09	g/L	0.41	mM
Glucose	2.00	g/L	11.1	mM
Maltose[1]	2.00	g/L	5.8	mM
Lactose[1]	2.00	g/L	5.8	mM
Tryptic digested Casein	10.00	g/L	10.0	g/L
Cysteine x HCl x H_2O	1.00	g/L	5.7	mM
Acetic acid[2]	0.9 / 1.7	g/L	15.0 / 28.3	mM
Propionic acid[2]	1.1 / 0.6	g/L	15.0 / 8.1	mM
n-Butyrate[2]	1.3 / 0.3	g/L	15.0 / 3.4	mM
Iso-Butyrate[2]	1.3 / 0.1	g/L	15.0 / 1.1	mM
n-Valeric acid[2]	1.5 / 0.1	g/L	15.0 / 1.0	mM
Iso-Valeric acid[2]	1.5 / 0.1	g/L	15.0 / 1.0	mM
Sodium lactate (60% w/v)[1]	10	mL/L	53.5	mM
Resazurin	1.00	mg/L	4.4	nM
CO_2 (v/v)	100	%	100	% gas phase
Vitamin solution[3]	0.1	mL/7 mL	(per Hungate tube)	

[1] These ingredients were omitted in the M2-B medium. [2] Short-chain fatty acid supplements for M2-A and M2-B. Left-hand values refer to the addition of single SCFAs to M2-A. Concentrations of combined SCFA supplementation to M2-B are given on the right. [3] Composition of vitamin solution see Table A1.

Table S3. Optical density and pH of cultivations for LCFA harvesting.

	Cultivation Conditions	OD_{600} Pre (1)	OD_{600} Pre (2)	OD_{600} Main	Mean ± SD of OD_{600}	pH Sample	pH Blank	ΔpH
8 h incubation	Acet	1.26	1.40	1.64	1.44 ± 0.19	5.17	6.51	1.34
	Prop	1.33	1.51	1.68	1.50 ± 0.17	5.18	6.50	1.32
	But	1.46	1.27	1.58	1.43 ± 0.16	5.20	6.53	1.33
	iBut	1.28	1.15	1.25	1.23 ± 0.07	5.73	6.58	0.85
	Val	1.71	1.70	1.77	1.73 ± 0.04	5.08	6.55	1.47
	iVal	1.11	1.11	1.27	1.16 ± 0.09	6.15	6.71	0.56

	Cultivation Conditions	OD_{600} Pre (1)	OD_{600} Pre (2)	OD_{600} Main	Mean ± SD of OD_{600}	pH Sample	pH Blank	ΔpH
24 h incubation	Acet	1.68	1.63	1.70	1.67 ± 0.04	5.19	6.57	1.38
	Prop	1.62	1.38	1.91	1.64 ± 0.26	4.93	6.50	1.57
	But	1.63	1.52	1.82	1.66 ± 0.15	4.92	6.53	1.61
	iBut	1.62	1.59	1.71	1.64 ± 0.06	5.17	6.66	1.49
	Val	1.73	1.67	1.92	1.77 ± 0.13	4.93	6.55	1.62
	iVal	1.15	1.64	1.82	1.54 ± 0.34	5.45	6.50	1.05

The cultivations are divided into Pre- and Main cultivations and with incubation time of 8 or 24 h. Pretreatments 1 and 2 represent the chronological order, followed by the main treatment. Mean and standard deviation (SD) of optical density were calculated for each condition (n=3). High SD at 24 h for iVal and Prop indicate deviation in inoculation composition or differences in adaptation time. Acidification is described as ΔpH and calculated as the difference between the pH at the start (blank) and the end of the incubation time (sample).

Table S4. Numbers of peptides and proteins determined by LC-MS/MS measurements.

Replicate	Acet	Prop	But	iBut	Val	iVal	
A	21,144	21,988	21,599	22,041	20,511	21,025	**Total peptides detected**
B	21,389	21,721	21,575	22,127	21,110	21,620	
C	21,660	22,023	22,160	21,693	20,872	20,667	
A	1,539	1,580	1,588	1,577	1,519	1,534	**Proteins detected**
B	1,550	1,580	1,566	1,578	1,554	1,543	
C	1,570	1,589	1,599	1,567	1,537	1,518	

Table S5. Cultivation parameters and relative amounts of trait specific long chain fatty acids.

Time	Condition	Acidification	Main OD	Mean OD	Branching			Chain length	
					anteiso	iso	linear	odd	even
8 h	Acet (C2 even)	1.34	1.64	1.44	38.7%	36.3%	25.0%	5.7%	94.3%
	Prop (C3 odd)	1.32	1.68	1.50	31.0%	34.3%	34.7%	17.2%	82.8%
	But (C4 even)	1.33	1.58	1.43	31.4%	30.8%	37.7%	5.9%	94.1%
	iBut (C4 odd)	0.85	1.25	1.23	26.7%	61.0%	12.3%	37.9%	62.1%
	Val (C5 odd)	1.47	1.77	1.73	30.8%	20.2%	49.0%	30.9%	69.1%
	iVal (C5 even)	0.56	1.27	1.16	37.0%	39.0%	24.0%	7.9%	92.1%
24 h	Acet (C2 even)	1.38	1.70	1.67	21.0%	56.2%	22.8%	5.7%	94.3%
	Prop (C3 odd)	1.57	1.91	1.64	31.5%	30.3%	38.2%	17.2%	82.8%
	But (C4 even)	1.61	1.82	1.66	29.6%	27.1%	43.4%	5.9%	94.1%
	iBut (C4 odd)	1.49	1.71	1.64	16.8%	71.3%	11.9%	37.9%	62.1%
	Val (C5 odd)	1.62	1.92	1.77	33.0%	19.4%	47.6%	30.9%	69.1%
	iVal (C5 even)	1.05	1.82	1.54	27.9%	53.0%	19.1%	7.9%	92.1%
**Pearson Correlation**		Mean OD vs. LCFA type			-0.33	-0.29	0.53	0.16	-0.16
		Main OD vs. LCFA type			-0.12	-0.38	0.54	-0.05	0.05
		Acidification vs. LCFA type			-0.21	-0.37	0.57	0.11	-0.11

The cultivation parameters like mean optical density of pre- and main culture (OD; n=3), the OD of the respective main culture and the acidification (n=3) were correlated with the LCFA type in the manner of Pearson to obtain the correlation coefficient (R).

Table S6. Percentile amount of long-chain fatty acids found in lipid membrane of *P. bryantii* B14.

	LCFA (branching)	Relative amount of LCFA [%] in conditions					
		Acet	Prop	But	iBut	Val	iVal
8 h	11-Me C12:0 (iso)	2.5	3.0	2.1	2.3	1.4	4.0
	12-Me C13:0 (iso)	4.6	4.1	4.7	25.8	4.4	4.0
	C14:0 (linear)	2.3	1.2	3.8	1.8	0.7	1.8
	13-Me C14:0 (iso)	19.7	18.7	16.1	19.6	12.4	25.5
	12-Me C14:0 (anteiso)	37.3	29.3	30.1	26.7	30.2	35.7
	C15:0 (linear)	-	11.5	0.1	-	25.4	2.7
	14-Me C15:0 (iso)	1.1	1.3	1.2	12.1	0.5	1.2
	C16:0 (linear)	18.3	10.3	24.0	4.7	9.1	9.0
	15-Me C16:0 (iso)	8.4	7.1	6.8	1.2	1.6	4.2
	14-Me C16:0 (anteiso)	1.4	1.7	1.3	-	0.6	1.4
	C17:0 (linear)	-	0.3	-	-	0.6	-
	C18:0 (linear)	4.4	11.4	9.9	5.8	13.2	10.5
24 h	11-Me C12:0 (iso)	2.5	2.0	1.3	1.3	1.2	3.1
	12-Me C13:0 (iso)	4.2	4.3	4.8	24.4	4.1	4.7
	C14:0 (linear)	2.0	0.9	3.4	1.6	0.4	2.6
	13-Me C14:0 (iso)	39.6	15.6	13.6	26.0	11.8	40.0
	12-Me C14:0 (anteiso)	19.3	29.7	27.9	15.9	31.8	26.6
	C15:0 (linear)	-	15.6	0.2	-	27.3	3.1
	14-Me C15:0 (iso)	1.4	1.4	1.5	17.6	0.9	1.3
	C16:0 (linear)	16.4	13.5	27.7	6.5	9.8	9.3
	15-Me C16:0 (iso)	8.6	7.0	6.0	2.0	1.5	4.0
	14-Me C16:0 (anteiso)	1.7	1.8	1.7	1.0	1.2	1.3
	C17:0 (linear)	-	1.7	-	-	1.1	-
	C18:0 (linear)	4.3	6.6	12.1	3.8	9.1	4.1

Prevotella was incubated with different SCFA, for incubation times at 8 and 24 h. The incubated SCFAs were iso-valeric acid (iVal),valeric acid (Val), iso-butyric acid (iBut), butyric acid (But), propionic acid (Prop) and acetic acid (Acet). The description of the long-chain fatty acids (LCFA) is described by the possible position of methylation, the number of linear carbon atoms behind the letter C and the corresponding type iso or anteiso in brackets.

Table S7. SCFA measurement of cultivation for LCFA analysis.

Treatment	C2 [mM]	C3 [mM]	C4I [mM]	C4 [mM]	C5I [mM]	C5 [mM]
Acet 8h_Blank	15.75	0.00	0.00	0.00	0.00	0.00
Acet 24h_Blank	14.76	0.00	0.00	0.00	0.00	0.00
Acet 8h	25.30	0.00	0.00	0.00	0.06	0.00
Acet 24h	28.70	0.00	0.00	0.00	0.08	0.00
Prop 8/24 h_Blank	0.00	14.53	0.00	0.04	0.00	0.00
Prop 8h	10.03	15.22	0.00	0.00	0.08	0.07
Prop 24h	16.05	14.80	0.00	0.00	0.07	0.06
But 8/24 h_Blank	0.00	0.10	0.00	15.50	0.00	0.00
But 8h	10.14	0.06	0.00	15.39	0.09	0.00
But 24h	16.79	0.00	0.00	15.06	0.10	0.00
iBut 8h_Blank	0.65	0.00	15.06	0.00	0.00	0.00
iBut 24h_Blank	0.46	0.00	15.20	0.00	0.00	0.00
iBut 8h	6.71	0.00	14.57	0.00	0.08	0.00
iBut 24h	14.48	0.00	14.33	0.00	0.09	0.00
Val 8/24 h_Blank	0.00	0.00	0.00	0.09	0.00	14.79
Val 8h	11.77	0.00	0.00	0.06	0.16	14.11
Val 24h	15.59	0.00	0.00	0.08	0.17	13.90
iVal 8h_Blank	0.72	0.00	0.00	0.00	14.44	0.00
iVal 24h_Blank	0.41	0.00	0.00	0.00	14.90	0.00
iVal 8h	6.93	0.00	0.00	0.00	14.20	0.00
iVal 24h	11.23	0.00	0.00	0.00	14.44	0.00

Acetic acid (C2), propionic acid (C3), butyric acid (C4), isobutyric acid (iC4), valeric acid (C5) and isovaleric acid (iC5) are displayed in mM for each approach in triplicate (n=3) with one blank (n=1). An inaccuracy of ±1.5 mM can be expected. The number of blanks in the table is depending if the cultivation for 8 or 24 h was performed in one or two runs.

Table S8. SCFA measurement from supernatant of cultivation for proteomic analysis.

Conditions	C2 [mM]	C3 [mM]	C4I [mM]	C4 [mM]	C5I [mM]	C5 [mM]
Acet 8h_Blank	17.30	0.00	0.00	0.00	0.03	0.00
Acet 8h_A	23.48	0.00	0.00	0.00	0.05	0.00
Acet 8h_B	23.19	0.00	0.00	0.00	0.06	0.00
Acet 8h_C	21.97	0.00	0.00	0.00	0.06	0.00
Prop 8h_Blank	0.00	14.99	0.00	0.00	0.02	0.00
Prop 8h_A	7.02	14.92	0.00	0.00	0.06	0.00
Prop 8h_B	6.81	14.92	0.00	0.00	0.05	0.00
Prop 8h_C	8.42	15.30	0.00	0.00	0.06	0.00
But 8h_Blank	0.00	0.00	0.00	15.01	0.00	0.00
But 8h_A	8.48	0.00	0.00	15.73	0.05	0.00
But 8h_B	8.70	0.00	0.00	13.82	0.06	0.00
But 8h_C	9.72	0.00	0.00	13.72	0.06	0.00
iBut 8h_Blank	0.00	0.00	17.03	0.03	0.00	0.00
iBut 8h_A	7.91	0.00	15.26	0.03	0.05	0.00
iBut 8h_B	7.98	0.00	0.55	0.00	0.06	0.00
iBut 8h_C	8.05	0.00	13.36	0.00	0.05	0.00
Val 8h_Blank	0.00	0.00	0.00	0.00	0.00	14.51
Val 8h_A	4.52	0.00	0.00	0.00	0.07	14.60
Val 8h_B	7.06	0.00	0.00	0.00	0.09	14.84
Val 8h_C	5.63	0.00	0.00	0.00	0.07	13.52
iVal 8h_Blank	0.00	0.00	0.00	0.00	14.31	0.06
iVal 8h_A	6.14	0.00	0.00	0.00	15.14	0.06
iVal 8h_B	6.11	0.00	0.00	0.00	13.11	0.00
iVal 8h_C	5.64	0.00	0.00	0.00	22.65	0.04

Acetic acid (C2), propionic acid (C3), butyric acid (C4), isobutyric acid (iC4), valeric acid (C5) and isovaleric acid (iC5) are displayed in milli molar for each approach in triplicate (n=3) with one blank (n=1). All concentrations can deviate ±1.5 mM. Outliers are written in red.

a

Table S9. Growth parameters of *P. bryantii* B_14 in M2-B.

Time [min]	Glucose (A) [mM]	Glucose (B) [mM]	pH (A)	pH (B)	OD_{600} (A)	OD_{600} (B)	Cell dry mass (A) [mg/mL]	Cell dry mass (B) [mg/mL]
0	15.96	18.88	6.83	6.88	0.31	0.35	0.00	-0.13
15	15.91	20.37	6.79	6.87	0.33	0.38	0.40	-0.20
35	16.97	18.43	6.75	6.87	0.38	0.44	0.00	0.20
50	17.76	18.17	6.83	6.88	0.43	0.50	0.20	0.00
65	16.37	15.99	6.71	6.86	0.48	0.58	0.07	-0.07
80	17.21	14.31	6.65	6.86	0.54	0.68	0.13	-0.07
95	15.31	14.93	6.63	6.71	0.61	0.77	0.07	0.00
110	13.85	14.38	6.57	6.68	0.71	0.90	0.27	0.13
125	12.70	10.66	6.36	6.60	0.80	1.22	0.33	0.10
140	11.72	9.59	6.32	6.59	1.09	1.51	0.20	0.00
150	11.00	7.65	6.16	6.40	1.19	1.87	0.30	0.40
160	11.38	7.48	6.09	6.37	1.38	2.18	0.50	0.56
170	8.46	5.87	5.99	6.26	1.80	1.98	0.72	0.72
180	7.74	3.71	5.90	6.20	2.19	1.96	0.48	0.48
190	6.52	3.14	5.72	6.05	2.16	2.39	0.56	0.88
210	3.33	1.17	5.56	5.79	2.30	2.62	0.96	0.96
226	1.27	0.19	5.44	5.62	2.46	2.73	0.96	1.04
240	0.39	0.08	5.44	5.58	2.71	2.93	1.04	0.88
255	0.10	0.07	5.43	5.51	3.06	3.22	1.09	1.38
270	0.08	0.07	5.42	5.48	3.06	2.89	1.09	1.23
290	0.06	0.06	5.43	5.45	2.96	2.95	0.36	1.09
335	0.04	0.06	5.38	5.42	3.07	2.94	1.23	1.45
395	0.00	0.03	5.39	5.40	2.82	2.98	1.01	1.52

Duplicates are indicated by (A) or (B), each n=1. The glucose is given in milli molar and was determined enzymatically. Optical density (OD) was measured at 600 nm wavelength. Dry mass was calculated by $mass_{(vessel)}$ - $mass_{(vessel + dry\ cells)}$.

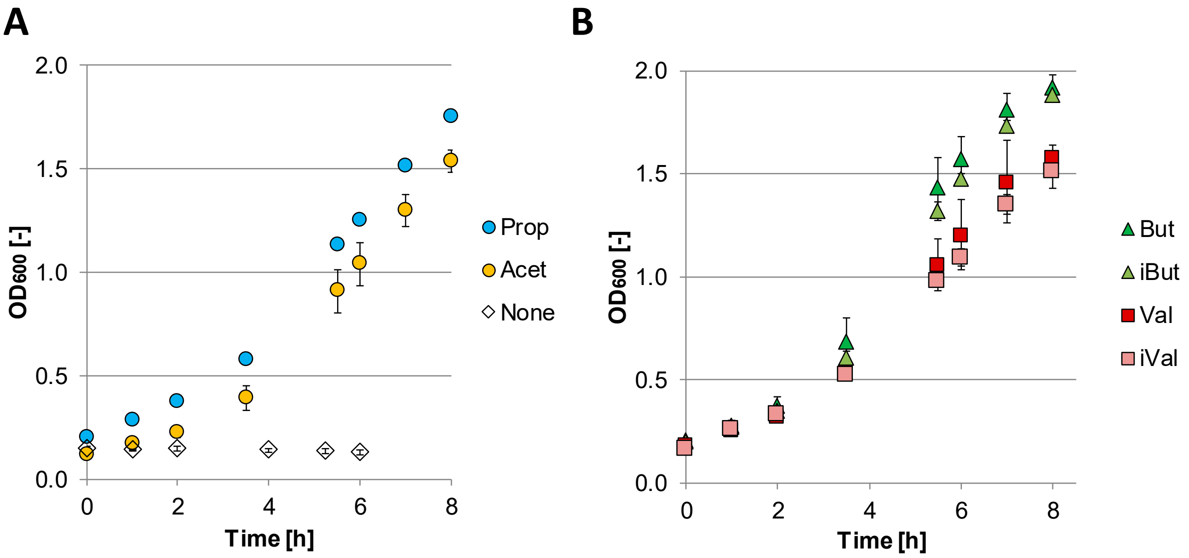

Figure S1. Cultivation of *P. bryantii* B14 in M2-A medium in the presence of various short-chain fatty acids for subsequent proteome analyses. Growth curve of *P. bryantii* culture (A) without SCFAs (None), acetic acid (Acet), propionic acid (Prop) and a second growth curve for better illustration (B) with butyric acid (But), iso-butyric acid (iBut), valeric acid (Val) and iso-valeric acid (iVal). Symbols represent the mean values of biological triplicates with errorbars as standard deviation.

Count of maximal abundant COGs per SCFA culture

	COGs Max count									
**Conditions**	**A**	**ADK**	**C**	**CH**	**CO**	**CP**	**D**	**DK**	**DM**	**DZ**
Acet	0	0	18	0	3	0	5	0	0	1
Prop	1	0	17	1	0	0	5	0	0	0
But	0	0	6	0	2	1	2	0	1	0
iBut	0	1	9	0	1	0	1	1	0	0
Val	0	0	14	0	2	0	4	0	0	0
iVal	0	0	22	0	3	0	7	0	0	0
**Conditions**	**E**	**EF**	**EG**	**EH**	**EJ**	**ET**	**EU**	**F**	**FG**	**FJ**
Acet	11	0	0	1	0	0	0	9	0	0
Prop	10	2	0	0	1	1	0	11	0	0
But	10	0	0	1	0	0	0	2	0	1
iBut	9	0	0	0	1	1	0	6	0	0
Val	21	0	0	0	0	0	1	21	0	0
iVal	54	0	1	3	0	0	1	24	1	3
**Conditions**	**FK**	**G**	**GK**	**GM**	**H**	**HP**	**HQ**	**I**	**IM**	**IQ**
Acet	0	24	0	1	13	0	0	10	0	5
Prop	0	13	0	0	11	0	0	1	1	0
But	0	37	0	1	12	0	0	8	0	1
iBut	0	43	0	2	3	0	0	2	0	0
Val	1	15	0	1	24	1	0	6	1	1
iVal	0	32	1	1	31	0	1	17	0	6
**Conditions**	**J**	**JM**	**K**	**KLT**	**KT**	**L**	**LT**	**M**	**MNU**	**MU**
Acet	11	0	7	0	1	9	1	27	1	0
Prop	36	0	25	1	2	27	0	25	0	1
But	2	1	9	0	1	13	0	35	0	1
iBut	3	0	7	0	1	2	0	19	0	1
Val	58	2	16	0	0	20	0	30	0	1
iVal	26	0	20	0	1	9	0	46	0	2
**Conditions**	**N**	**NU**	**O**	**OU**	**P**	**Q**	**S**	**T**	**U**	**V**
Acet	1	1	13	0	18	3	65	4	0	4
Prop	0	0	13	0	7	2	46	7	5	7
But	0	0	2	0	16	2	44	5	5	6
iBut	1	0	2	0	11	3	32	2	1	2
Val	0	0	19	0	10	1	64	8	2	5
iVal	1	1	13	2	9	4	91	8	2	2
**Color code:**	0	1	20	50	90					

count of the **maximal** abundant proteins, sorted by COGs and condition

**A**	RNA processing and modification
**B**	Chromatin Structure and dynamics
**C**	Energy production and conversion
**D**	Cell cycle control and mitosis
**E**	Amino Acid metabolis and transport
**F**	Nucleotide metabolism and transport
**G**	Carbohydrate metabolism and transport
**H**	Coenzyme metabolism
**I**	Lipid metabolism
**J**	Tranlsation
**K**	Transcription
**L**	Replication and repair
**M**	Cell wall/membrane/envelop biogenesis
**N**	Cell motility
**O**	Post-translational modification, protein turnover, chaperone functions
**P**	Inorganic ion transport and metabolism
**Q**	Secondary Structure
**R**	General Functional Prediction only
**S**	Function Unknown
**T**	Signal Transduction
**U**	Intracellular trafficing and secretion
**V**	Defense mechanism
**Y**	Nuclear structure
**Z**	Cytoskeleton

Figure S2. Count of maximal abundant COGs per SCFA supplementation.
Proteins which were maximal expressed among all cultures were counted and grouped by their functionality using the cluster of orthologous groups (COGs). Colors indicate the number of proteins with maximal abundance within a COG and cultivation condition. The color code is illustrated at the bottom of the graph. COGs are explained on the right side. Proteins with multiple COG assignations can also be found.

Count of least abundant COGs per SCFA culture

COGs Min count

Conditions	A	ADK	C	CH	CO	CP	D	DK	DM	DZ
Acet	0	0	25	1	0	0	6	1	0	0
Prop	0	0	3	0	5	0	3	0	0	0
But	0	0	7	0	1	0	5	0	0	0
iBut	0	0	16	0	2	1	5	0	0	0
Val	0	1	24	0	2	0	2	0	0	0
iVal	1	0	11	0	1	0	3	0	1	1

Conditions	E	EF	EG	EH	EJ	ET	EU	F	FG	FJ
Acet	38	0	1	1	0	1	2	15	0	2
Prop	8	0	0	0	1	0	0	9	0	0
But	14	0	0	0	0	0	0	10	0	0
iBut	21	0	0	1	0	1	0	17	0	1
Val	22	0	0	2	1	0	0	13	1	1
iVal	12	2	0	1	0	0	0	9	0	0

Conditions	FK	G	GK	GM	H	HP	HQ	I	IM	IQ
Acet	0	41	0	2	22	1	1	8	1	2
Prop	0	15	0	0	12	0	0	1	0	0
But	0	11	0	1	10	0	0	10	0	2
iBut	0	12	1	0	15	0	0	6	1	1
Val	0	56	0	2	19	0	0	14	0	5
iVal	1	29	0	1	16	0	0	5	0	3

Conditions	J	JM	K	KLT	KT	L	LT	M	MNU	MU
Acet	33	1	34	0	1	25	0	57	0	0
Prop	3	0	7	0	1	6	0	21	0	2
But	10	0	3	0	1	3	0	16	0	1
iBut	41	1	10	0	0	9	0	25	0	0
Val	5	1	9	0	1	11	0	37	1	3
iVal	44	0	21	1	2	26	1	26	0	0

Conditions	N	NU	O	OU	P	Q	S	T	U	V
Acet	0	0	16	1	15	5	105	15	3	4
Prop	1	1	8	0	8	1	41	3	0	4
But	0	0	6	0	7	1	37	4	1	1
iBut	0	1	14	1	6	1	44	4	5	6
Val	2	0	6	0	20	5	55	5	5	5
iVal	0	0	12	0	15	2	60	3	1	6

Color Code:	0	1	20	50	100

count of the **minimal** abundant proteins, sorted by COGs and condition

**A**	RNA processing and modification
**B**	Chromatin Structure and dynamics
**C**	Energy production and conversion
**D**	Cell cycle control and mitosis
**E**	Amino Acid metabolis and transport
**F**	Nucleotide metabolism and transport
**G**	Carbohydrate metabolism and transport
**H**	Coenzyme metabolism
**I**	Lipid metabolism
**J**	Tranlsation
**K**	Transcription
**L**	Replication and repair
**M**	Cell wall/membrane/envelop biogenesis
**N**	Cell motility
**O**	Post-translational modification, protein turnover, chaperone functions
**P**	Inorganic ion transport and metabolism
**Q**	Secondary Structure
**R**	General Functional Prediction only
**S**	Function Unknown
**T**	Signal Transduction
**U**	Intracellular trafficing and secretion
**V**	Defense mechanism
**Y**	Nuclear structure
**Z**	Cytoskeleton

Figure S3. Count of least abundant COGs per culture conditions.
Proteins which were least expressed among all cultivation conditions were counted and grouped by their functionality using the cluster of orthologous groups (COGs). Colors indicate the number of proteins with minimal abundance within a COG and SCFA. The color code is illustrated at the bottom of the graph. COGs are explained on the right side. Proteins with multiple COG assignations can also be found.

Name (Uniprot ID)	Acet	Prop	But	iBut	Val	iVal	Compound	EC
							L-Glutamate	
Glutamate--tRNA ligase (D8DU35)	1.00	1.03	0.92	0.85	1.27	0.97	↓	6.1.1.17
							L-Glutamyl trans (Glu)	
Glutamyl-tRNA reductase (A0A1H9I9M5)	1.00	0.71	1.16	0.83	1.62	1.01	↓	1.2.1.70
							Glutamate-1-semialdehyde	
Glutamate-1-semialdehyde 2,1-aminomutase (D8DTV2)	1.00	0.82	0.97	0.75	1.08	1.15	↓	5.4.3.8
							5-Amino-levulinate	
Porphobilinogen synthase (D8DTV1)	1.00	0.76	0.97	0.74	1.11	1.20	↓	4.2.1.24
							Porphobilinogen	
Hydroxymethylbilane synthase (D8DTU9)	1.00	0.73	0.83	0.76	1.29	0.89	↓	2.5.1.61
							Hydroxymethybilane	
Uroporphyrinogen-III synthase (D8DT34)	1.00	1.24	1.20	1.02	1.18	1.09	↓	4.2.1.75
							Uroporphybilinogen III	
Uroporphyrinogen decarboxylase (D8DTU7)	1.00	0.69	0.77	0.69	0.86	0.96	↓	4.1.1.37
							Coproporphyrinogen III	
Coproporphyrinogen-III oxidase (D8DUC6)	1.00	0.88	1.10	0.75	1.50	1.29	↓	1.3.98.3
							Protoporphyrinogen XI	
Protoporphyrinogen oxidase (D8DUC7)	1.00	0.87	1.19	0.87	1.93	0.75	↓	1.3.3.4
							Protoporphyrin XI	
							Hydroxymethybilane	
**Spontaneous reaction**							↓	-
							Uroporphybilinogen I	
Uroporphyrinogen decarboxylase (D8DTU7)	1.00	0.69	0.77	0.69	0.86	0.96	↓	1.3.3.15
							Coproporphyrinogen I / Uroporphybilinogen I	

Color code: 0.6 | 0.8 | 1.0 | 1.2 | 1.5 | 2.0

Figure S4. Porphyrin synthesis pathway in *P. bryantii* B14 with enzyme abundances. Protein abundances standardized by protein abundance of the Acet culture. Enzyme names are given with the Uniprot ID in brackets and the EC number (in the right side). A side reaction of the pathway is illustrated in the lower part. Chemical compounds are written in italic. Color code is given below the figure.

Amino acid biosynthesis

Name (Uniprot ID)	Acet	Prop	But	iBut	Val	iVal
3-isopropylmalate dehydratase large subunit (A0A1H9CVK2)	1.00	2.03	1.53	1.38	1.27	2.74
3-isopropylmalate dehydratase small subunit (D8DZ05)	1.00	1.75	1.60	1.43	1.50	2.36
Argininuccinate synthase (D8DU50)	1.00	1.57	1.41	1.08	1.45	2.33
Gamma-glutamyl Phosphate reductase (D8DU57)	1.00	1.50	1.51	1.34	1.30	2.04
Ribulose-phosphate 3-epimerase (D8DZK5)	1.00	1.24	1.28	0.96	1.52	2.57
Ketol-acid reductoisomerase (D8DZE6)	1.00	1.56	1.44	1.10	1.56	1.90
Aconitase (D8DYN3)	1.00	1.62	1.44	1.32	1.42	1.69
Acetylornithine aminotransferase (D8DU52)	1.00	1.29	1.16	1.01	1.05	2.87
Anthranilate Phosphoribosyltransferase (D8DZK7)	1.00	1.45	1.41	1.45	1.06	1.94
Anthranilate synthase component 1 (A0A1H8ZZV6)	1.00	1.78	1.49	1.39	0.65	1.93
Histidine biosynthesis bifunctional protein (D8DY88)	1.00	1.62	1.24	1.27	1.33	1.47
Glutamate synthase (D8DY67)	1.00	1.53	1.45	1.37	1.11	1.38
S-ribosylhomocysteine lyase (D8DYD4)	1.00	0.96	1.17	1.16	1.57	1.93
Glutamate synthase large subunit (D8DTH3)	1.00	1.71	1.81	1.64	0.00	1.30
Putative oxaloacetate decarboxylase (D8DUR7)	1.00	1.38	1.21	1.05	1.35	1.30
Probable Phosphoglycerate mutase (D8DUV1)	1.00	0.95	1.09	0.94	1.75	1.53
3-phosphhikimate 1-carboxyvinyltransferase (D8DVT2)	1.00	1.14	1.14	1.09	1.12	1.74
Isocitrate dehydrogenase (A0A1H9LFP7)	1.00	1.38	1.16	1.17	0.98	1.47
Aspartokinase (D8DY90)	1.00	1.28	1.15	1.02	1.32	1.26
Tryptophan synthase (D8DUX1)	1.00	1.34	1.36	1.26	0.79	1.24
Methionine synthase (A0A1H9FCA0)	1.00	1.36	1.25	1.12	1.10	1.13
Acetolactate synthase (D8DZE3)	1.00	1.31	1.01	1.42	0.87	1.26
ATP-dependent 6-phosphofructokinase (D8DW45)	1.00	1.12	1.00	1.09	1.13	1.49
Phosphoribosylformimino-5-aminoimidazole carboxamide ribotide isomerase (D8DY86)	1.00	1.07	0.93	1.22	1.14	1.43
Carboxynorspermidine dehydrogenase (D8DYF8)	1.00	1.27	1.00	1.00	1.18	1.30
D-3-phosphoglycerate dehydrogenase (D8DWG1)	1.00	1.23	1.04	1.11	1.22	1.12
Argininsuccinate lyase (D8DXE8)	1.00	1.35	1.09	1.19	0.87	1.16
Phospherine phosphatase (D8DTT3)	1.00	1.27	1.13	0.97	1.15	1.14
Fructose-1,6-bisphosphate aldolase, class II (D8E032)	1.00	1.05	0.93	0.85	1.36	1.44
Triosephosphate isomerase (D8DVS6)	1.00	0.90	0.77	0.87	1.47	1.55
Anthranilate synthase component 2 (D8DZL3)	1.00	1.20	0.82	0.99	0.66	1.76
Histidine biosynthesis bifunctional protein (D8DWS5)	1.00	1.05	1.00	0.78	1.45	0.97
5'-methylthioadenine/S-adenylhomocysteine nucleidase (D8DVQ8)	1.00	0.74	0.75	1.15	1.07	1.53
Homerine O-acetyltransferase (D8DYN0)	1.00	0.99	0.87	0.93	1.31	1.03
ATP Phosphoribosyltransferase (D8DYW0)	1.00	1.10	0.93	1.00	1.12	0.88
S-adenylmethionine synthase (D8DT36)	1.00	0.84	1.01	0.89	1.23	1.05
Bifunctional aspartokinase/homerine dehydrogenase I (D8DYS1)	1.00	0.99	0.92	0.80	1.52	0.73
Ribose-phosphate pyrophosphokinase (A0A1H9J4B5)	1.00	0.98	0.84	0.73	1.49	0.80
3-dehydroquinate dehydratase (D8DT38)	1.00	1.05	0.88	0.83	0.94	1.12
Phospherine aminotransferase (A0A1H9D5W0)	1.00	0.97	1.02	0.89	0.89	0.99
Imidazole glycerol Phosphate synthase subunit (D8DY87)	1.00	0.82	0.73	0.94	0.84	1.29
Threonine ammonia-lyase (D8DZH1)	1.00	0.89	0.88	0.87	0.74	0.89
Glutamate--ammonia ligase (D8DTI2)	1.00	0.72	0.77	0.76	1.33	0.58
Imidazole glycerol Phosphate synthase subunit (D8DY85)	1.00	0.79	0.62	0.82	0.78	1.10
Meso-diaminopimelate D-dehydrogenase (D8DYD8)	1.00	0.85	0.74	0.80	0.84	0.83
L-threonine aldolase (D8DTJ8)	1.00	0.73	0.78	0.68	0.96	0.73
Ribose-5-phosphate isomerase B (D8DYQ0)	1.00	0.75	0.76	0.91	0.58	0.78
N-(5'-phosphoribosyl)anthranilate isomerase (D8DZK9)	1.00	0.63	0.57	0.67	0.83	0.69
Glyceraldehyde-3-phosphate dehydrogenase (D8DZJ1)	1.00	0.70	0.48	0.58	0.89	0.75
**Color code:**	0.00	0.2	1.0	1.5	2.0	2.5

Figure S5. Heat map of enzymes from the amino acid synthesis pathway (ko01230).
Label-free quantification (LFQ) values of the amino acid metabolism from KEGG (ko01230) are related and standardized by the Acet culture and represented the average abundance in form of a heatmap, see color code below. Many enzymes also overlap with The UniprotKB is given in brackets after the protein name.

SIMPER Analysis

Name (Uniport ID)	Acet	Prop	But	iBut	Val	iVal
Phosphoenolpyruvate carboxykinase (ATP) (D8DZS4)	1.00	1.37	1.23	1.21	1.35	2.80
L-fucose isomerase (D8DSY3)	1.00	1.54	1.38	1.31	0.95	2.24
GGGtGRT protein (D8DZB7)	1.00	1.71	1.48	1.33	1.15	1.66
60 kDa chaperonin (D8DZI6)	1.00	1.30	1.06	0.99	1.52	1.77
Iron complex outermembrane recepter protein (D8DXY3)	1.00	1.13	1.40	1.33	0.75	1.19
Fructose-1,6-bisphosphate aldolase, class II (D8E032)	1.00	1.05	0.93	0.85	1.36	1.44
Formate C-acetyltransferase (D8E078)	1.00	1.16	1.01	0.98	1.17	1.27
Triosephosphate isomerase (D8DVS6)	1.00	0.90	0.77	0.87	1.47	1.55
Phosphoglycerate kinase (D8DY78)	1.00	1.17	0.91	0.98	1.19	1.24
Elongation factor Tu (A0A1H9JXP7)	1.00	1.07	0.78	0.97	0.90	1.65
Outer membrane protein (D8DXY1)	1.00	0.94	1.31	1.19	0.67	1.12
TPR domain protein (D8E017)	1.00	0.70	0.98	1.41	0.27	1.81
3-oxoacyl-[acyl-carrier-protein] synthase 2 (D8DXQ1)	1.00	1.06	0.86	0.93	0.90	1.40
Pyruvate, Phosphate dikinase (D8DWY7)	1.00	1.15	0.84	0.88	1.18	1.03
OmpA family protein (D8DZE1)	1.00	1.00	0.91	0.83	1.09	1.17
2-dehydro-3-deoxygluconokinase (D8DWR0)	1.00	0.92	0.96	1.13	0.83	0.93
Polyribonucleotide nucleotidyltransferase (A0A1H9C5Q4)	1.00	0.98	0.86	0.79	0.94	1.17
50S ribosomal protein L7/L12 (A0A1H9JYE1)	1.00	0.72	0.79	0.71	1.02	1.21
Phosphorylase family protein (D8DYI9)	1.00	0.95	0.72	0.90	0.66	1.17
IPT/TIG domain-containing protein (D8DY17)	1.00	1.12	0.83	1.48	0.40	0.45
Putative lipoprotein (D8DWD4)	1.00	0.74	0.87	0.88	0.73	0.66
Uncharacterized protein (A0A1H9L390)	1.00	0.68	0.71	0.69	0.87	0.83
Phosphoribylaminoimidazolecarboxamide formyltransferase (D8DZZ6)	1.00	0.63	0.69	0.61	1.00	0.69
Glyceraldehyde-3-phosphate dehydrogenase (D8DZJ1)	1.00	0.70	0.48	0.58	0.89	0.75
Putative tetratricopeptide repeat domain protein (D8DZ11)	1.00	0.44	0.58	0.88	0.45	0.92
Pyridoxal 5'-phosphate synthase subunit PdxS (D8DSV8)	1.00	0.39	0.41	0.43	1.49	0.29
Putative lipoprotein (D8DTP2)	1.00	0.36	0.24	0.48	1.67	0.17
Cationic outer membrane protein OmpH (D8DT44)	1.00	0.33	0.41	0.72	0.24	0.64
Enolase (D8E0G3)	1.00	0.36	0.35	0.38	0.83	0.22
Imelysin (D8DZR1)	1.00	0.28	0.29	0.30	1.09	0.16
Flavodoxin (D8DZB4)	1.00	0.34	0.36	0.38	0.80	0.20
Iron complex outermembrane recepter protein (A0A1H9ICS8)	1.00	0.31	0.36	0.29	0.71	0.31
**Color code:**	0.20	0.50	1.00	1.50	2.00	2.50

Figure S6. Similar percentage (SIMPER) analyzed proteins with >1% contribution.
The listed proteins contribute with at least 1% to the differential plotting in the PCO plot of Figure 1. Label-free quantification (LFQ) values are related and standardized by the Acet culture and represented the average abundance in form of a heatmap, see color code below. The UniprotKB is followed in brackets after the protein name.

Outer membrane proteins

Name (Uniprot ID)	Acet	Prop	But	iBut	Val	iVal
Outer membrane protein beta-barrel domain-containing protein (D8DWQ2)	1.00	1.70	3.03	4.23	0.90	2.22
Starch-binding associating with outer membrane (A0A1H9C300)	1.00	1.88	1.42	1.47	2.55	2.43
Outer membrane receptor proteins, mostly Fe transport (D8DZ53)	1.00	1.32	1.18	1.76	0.00	2.57
Outer membrane protein (D8DXY2)	1.00	1.31	1.64	1.43	0.86	1.48
Periplasmic chaperone for outer membrane proteins Skp (D8DSW6)	1.00	1.46	1.32	1.10	1.40	1.30
Outer membrane protein beta-barrel domain-containing protein (D8DZ08)	1.00	1.12	1.32	1.13	1.02	1.45
Outer membrane protein (D8DT53)	1.00	1.34	0.94	1.88	0.92	0.64
Periplasmic chaperone for outer membrane proteins Skp (D8DT43)	1.00	0.99	0.89	0.95	0.88	1.66
Outer membrane protein TolC (D8DWK0)	1.00	0.81	0.92	1.06	1.37	1.19
Outer membrane protein beta-barrel domain-containing protein (D8DWZ7)	1.00	1.02	0.87	1.07	1.00	1.33
Outer membrane lipoprotein-sorting protein (A0A1H9GE28)	1.00	1.00	1.06	0.98	0.97	1.21
Outer membrane protein (D8DXY1)	1.00	0.94	1.31	1.19	0.67	1.12
Starch-binding associating with outer membrane (A0A1H9E645)	1.00	1.10	0.90	1.03	1.09	0.91
Major outer membrane protein OmpA (D8DWD3)	1.00	1.06	1.12	1.00	1.09	0.75
TonB-linked outer membrane protein, SusC/RagA family (A0A1H9B8Y5)	1.00	0.98	1.13	1.48	0.53	0.89
Outer membrane protein (D8DZK0)	1.00	0.94	1.09	1.26	0.61	1.02
Outer membrane protein beta-barrel domain-containing protein (D8DZT1)	1.00	0.57	1.08	1.12	0.67	1.41
Outer membrane receptor proteins, mostly Fe transport (A0A1H9DV15)	1.00	1.16	1.28	0.99	0.67	0.74
Outer membrane receptor proteins, mostly Fe transport (A0A1H9CY22)	1.00	0.88	1.15	0.93	0.87	0.98
Outer membrane protein beta-barrel family protein (A0A1H9HV83)	1.00	1.06	1.15	1.14	0.71	0.75
Outer membrane protein beta-barrel domain-containing protein (D8DUD8)	1.00	0.94	0.94	0.86	0.82	1.23
Outer membrane receptor proteins, mostly Fe transport (D8DTP4)	1.00	0.98	0.99	0.84	0.82	1.08
Outer membrane protein OmpA (A0A1H9DD30)	1.00	0.87	0.87	0.93	1.08	0.92
Outer membrane protein assembly factor BamA (D8DWS2)	1.00	0.80	0.92	0.80	1.14	1.00
Outer membrane efflux protein (D8DYZ4)	1.00	0.90	0.76	0.88	0.67	1.27
Outer membrane protein, cobalt-zinc-cadmium efflux system (A0A1H9FFT0)	1.00	1.07	0.97	1.06	0.52	0.87
TonB-linked outer membrane protein, SusC/RagA family (A0A1H9C2R2)	1.00	0.87	0.86	0.81	0.98	0.91
Outer membrane lipoprotein carrier protein LolA (D8DTI7)	1.00	0.89	0.86	0.85	0.88	0.94
Efflux transporter, outer membrane factor (OMF) lipoprotein, NodT family (D8DVS0)	1.00	0.82	0.67	1.03	0.74	1.13
TonB-linked outer membrane protein, SusC/RagA family (A0A1H9LDK0)	1.00	0.84	0.81	0.85	0.94	0.82
Outer membrane protein beta-barrel family protein (A0A1H8Z174)	1.00	0.80	0.77	0.79	0.75	1.02
Outer membrane protein SusC (D8E073)	1.00	0.87	1.16	1.11	0.00	0.81
Starch-binding associating with outer membrane (D8DV05)	1.00	0.86	1.23	1.07	0.26	0.26
Outer membrane protein beta-barrel domain-containing protein (D8DYY2)	1.00	0.63	0.65	0.76	0.51	0.98
Outer membrane protein beta-barrel domain-containing protein (D8DVA3)	1.00	0.64	0.92	0.82	0.52	0.62
Outer membrane autotransporter barrel domain-containing protein (D8DU39)	1.00	0.76	0.70	0.88	0.46	0.69
Cationic outer membrane protein OmpH (D8DT45)	1.00	0.67	0.66	0.86	0.37	0.88
Outer membrane receptor proteins, mostly Fe transport (D8E0H5)	1.00	0.50	0.64	0.81	0.69	0.70
Outer membrane protein transport protein (Ompp1/fadl/todx) (D8DTK4)	1.00	0.81	0.62	0.70	0.58	0.61
Starch-binding associating with outer membrane (D8DUK8)	1.00	0.79	0.58	0.95	0.47	0.38
Outer membrane protein beta-barrel domain-containing protein (D8DXU0)	1.00	0.58	0.42	0.54	0.72	0.41
TonB-dependent outer membrane receptor (D8DUK7)	1.00	0.61	0.47	0.75	0.44	0.30
TonB-linked outer membrane receptor P92 (D8DTK2)	1.00	0.54	0.46	0.53	0.51	0.45
Cationic outer membrane protein OmpH (D8DT44)	1.00	0.33	0.41	0.72	0.24	0.64
Outer membrane protein beta-barrel family protein (D8E007)	1.00	0.00	0.00	0.53	1.42	0.00
TonB-dependent outer membrane receptor (D8DXZ1)	0.00	1.69	0.00	0.00	0.00	1.00
Outer membrane receptor for ferrienterochelin and colicins (D8E003)	1.00	0.21	0.16	0.23	0.83	0.10
Outer membrane protein beta-barrel domain-containing protein (D8DW23)	1.00	0.00	0.00	0.37	1.07	0.00
Starch-binding associating with outer membrane (D8DVB5)	0.00	1.00	0.00	1.07	0.00	0.00
Outer membrane receptor proteins, mostly Fe transport (D8DSX8)	1.00	0.00	0.00	0.00	0.45	0.00
Starch-binding associating with outer membrane (A0A1H8ZVZ9)	0.00	0.00	0.00	1.00	0.00	0.00
**Color code:**	0.00	0.2	1.0	1.5	2.0	2.5

Figure S7. Heat map of most abundant outer membrane proteins.
Label-free quantification (LFQ) values are related and standardized by the Acet culture and represent the average abundance in form of a heatmap, see color code below. In cases where Acet is zero, proteins from the cultivation condition with the lowest abundance were set to one. Proteins written in red font indicate proteins involved in iron transport.The UniprotKB is given in brackets after the protein name.

Acet and Val specific proteins

COG	Name (Uniprot ID)	Acet	Prop	But	iBut	Val	iVal
M	Putative integrin, alpha subunit (D8DW34)	1.00	0.20	0.34	0.61	3.07	0.30
M	Acyltransferase (D8DYW5)	1.00	0.63	0.71	0.67	0.94	0.61
M	Biosynthetic peptidoglycan transglycylase (A0A1H9D129)	1.00	0.86	0.80	0.80	1.00	0.83
M	Outer membrane protein assembly factor BamA (D8DWS2)	1.00	0.80	0.92	0.80	1.14	1.00
M	Outer membrane protein beta-barrel domain-containing protein (D8DW23)	1.00	0.00	0.00	0.37	1.07	0.00
M	Outer membrane protein beta-barrel domain-containing protein (D8DXU0)	1.00	0.58	0.42	0.54	0.72	0.41
M	Outer membrane protein OmpA (A0A1H9DD30)	1.00	0.87	0.87	0.93	1.08	0.92
M	Peptidyl-prolyl cis-trans isomerase (A0A1H8YU86)	1.00	0.75	0.59	0.74	0.78	0.77
M	Putative membrane protein (D8DVX1)	1.00	0.72	0.69	0.85	0.87	0.66
M	Uncharacterized protein (A0A1H9FPB8)	1.00	0.98	0.98	0.93	1.29	0.93
P	Fur family transcriptional regulator, peroxide stress response regulator (D8DTP6)	1.00	0.31	0.08	0.19	1.64	0.07
P	CarboxypepD (D8E0I3)	1.00	0.60	0.80	0.86	0.94	0.82
P	Ferrous iron transport protein B (D8DT71)	1.00	0.87	0.84	0.76	1.09	0.67
P	Iron complex outermembrane recepter protein (A0A1H9ICS8)	1.00	0.31	0.36	0.29	0.71	0.31
P	Outer membrane receptor for ferrienterochelin and colicins (D8E003)	1.00	0.21	0.16	0.23	0.83	0.10
P	Outer membrane receptor proteins, mostly Fe transport (D8DSX8)	1.00	0.00	0.00	0.00	0.45	0.00
P	TonB-dependent Receptor Plug Domain (A0A1H8ZS43)	1.00	0.51	0.45	0.63	0.91	0.44
P	TonB-linked outer membrane protein, SusC/RagA family (A0A1H9C2R2)	1.00	0.87	0.86	0.81	0.98	0.91
P	TonB-linked outer membrane protein, SusC/RagA family (A0A1H9LDK0)	1.00	0.84	0.81	0.85	0.94	0.82
P	Uncharacterized protein (D8DZ73)	1.00	0.59	0.78	0.53	1.33	0.64
P	Zinc ABC transporter, ATP-binding protein ZnuC (D8DT91)	1.00	0.75	0.75	0.70	0.90	0.76
S	Imelysin (D8DZR1)	1.00	0.28	0.29	0.30	1.09	0.16
S	AAA domain-containing protein, putative AbiEii toxin (D8DVG6)	1.00	0.90	0.95	0.92	1.03	0.87
S	ATP:cob(I)alamin adenyltransferase (D8DZF8)	1.00	0.65	0.52	0.63	0.75	0.62
S	DedA family protein (D8DV79)	1.00	0.43	0.55	0.52	0.61	0.43
S	FeS assembly SUF system protein (D8DU67)	1.00	0.60	0.70	0.72	0.98	0.71
S	Glutamate-ammonia ligase (D8DTI2)	1.00	0.72	0.77	0.76	1.33	0.58
S	HmuY protein (D8E002)	1.00	0.25	0.21	0.29	0.98	0.13
S	LruC domain-containing protein (D8DZM3)	1.00	0.39	0.54	0.47	0.92	0.66
S	Membrane protein YfhO (D8DVQ7)	1.00	0.97	0.95	0.82	1.10	0.82
S	PepSY-associated TM helix domain protein (D8DUK5)	1.00	0.51	0.53	0.00	0.58	0.00
S	Peptidase, M16 family (D8DUF2)	1.00	0.35	0.60	0.46	0.71	0.40
S	Predicted ATP-binding protein involved in virulence (A0A1H9KT98)	1.00	0.85	0.90	0.87	0.96	0.84
S	Putative lipoprotein (D8DSX9)	1.00	0.00	0.26	0.25	0.71	0.00
S	Putative lipoprotein (D8DZ94)	1.00	0.53	0.55	0.61	0.70	0.58
S	Putative lipoprotein (D8E004)	1.00	0.31	0.24	0.29	1.04	0.14
S	Putative membrane protein (D8DZ34)	1.00	0.00	0.00	0.00	1.76	0.00
S	RloB-like protein (A0A1H9GW48)	1.00	0.85	0.91	0.00	0.92	0.00
S	RNA polymerase Rpb6 (D8DYA9)	1.00	0.96	0.99	0.75	1.04	0.81

**Color code:**	0.00	0.2	0.5	1.0	1.5	2.0

Figure S8. Acet and Val culture specific proteins.
Label-free quantification (LFQ) values are related and standardized by the Acet culture and represent the average abundance in form of a heatmap, see color code below. In cases where Acet is zero, proteins from cultivation condition with the lowest abundance were set to one. Proteins written with a red font indicate proteins involved in iron transport. The UniprotKB is given in brackets after the protein name. Proteins are sorted by the major clusters of orthologous groups (COGs). COG functions: membrane and cell wall assembly (M), inorganic transport (P) and unknown function (S).

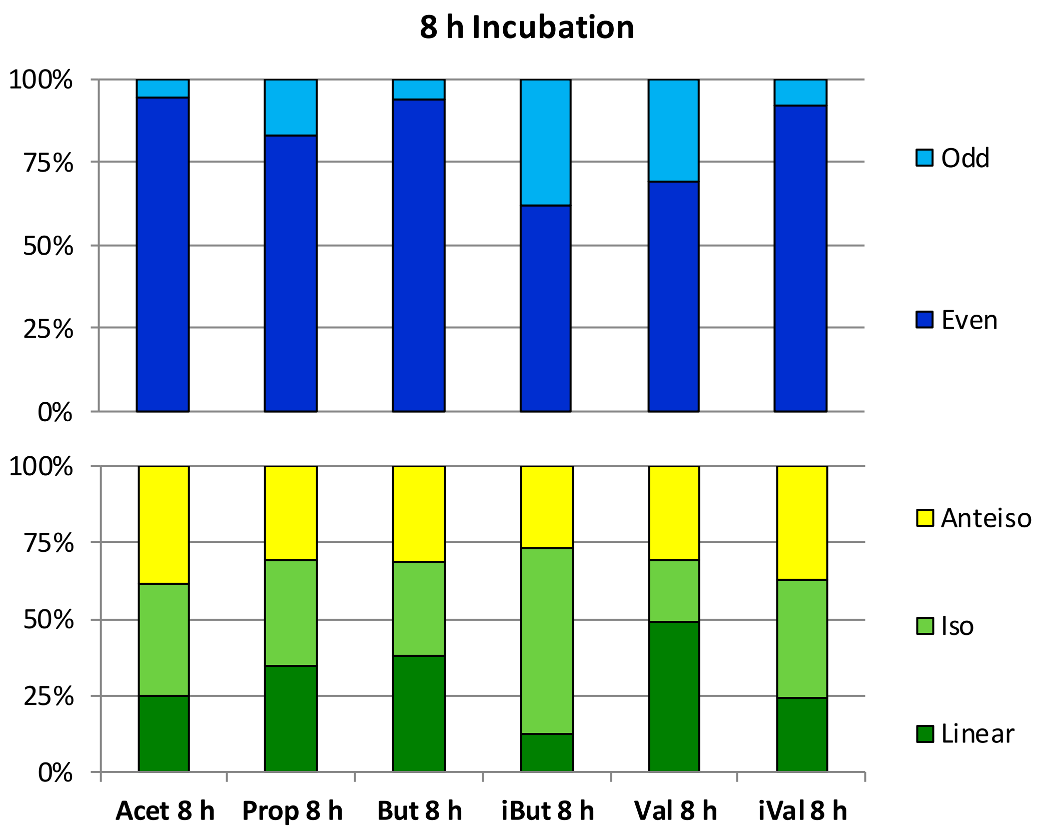

Figure S9. Percentile distribution of long-chain fatty acids (LCFA) by traits of chain length and branching structure after 8 h incubation. The distribution of the trait with odd or even number of the straight chain length of carbon atoms is shown in bluish colors (**upper**). The chain length trait divides the fatty acid length in even for Acet, But and iVal and odd for Prop, iBut and Val. The distribution of non-branched (linear), iso- or anteiso methylated long-chain fatty acids are in yellow-greenish colors (**lower**). SCFAs are grouped by the branching trait in linear for Acet, Prop, But, Val and in iso for iBut and iVal. The two traits are calculated by the sum of the percentile appearance in the LCFA-profile.

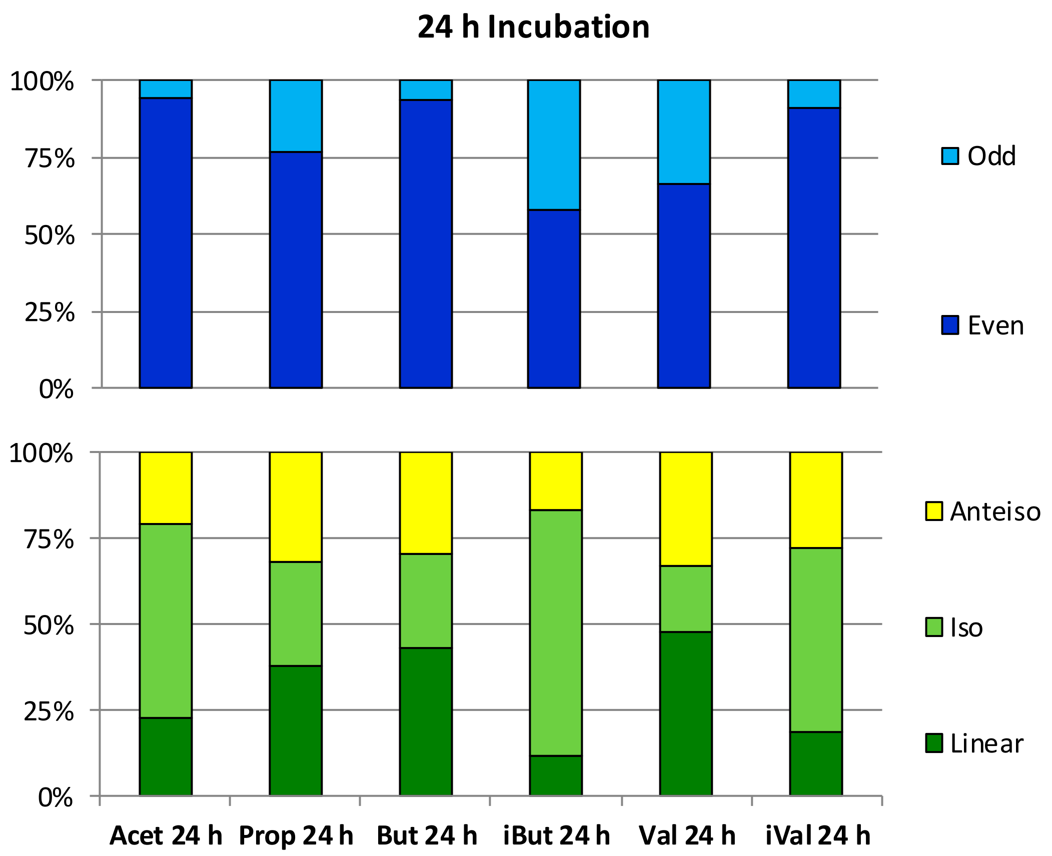

Figure S10. Percentile distribution of long-chain fatty acids (LCFA) by traits of chain length and branching structure after 24 h incubation. The distribution of the trait with odd or even number of the straight chain length of carbon atoms is shown in bluish colors (**upper**). The chain length trait divides the fatty acid length in even for Acet, But and iVal and odd for Prop, iBut and Val. The distribution of non-branched (linear), iso- or anteiso methylated long-chain fatty acids are in yellow-greenish colors (**lower**). SCFAs are grouped by the branching trait in linear for Acet, Prop, But, Val and in iso for iBut and iVal. The two traits are calculated by the sum of the percentile appearance in the LCFA-profile.

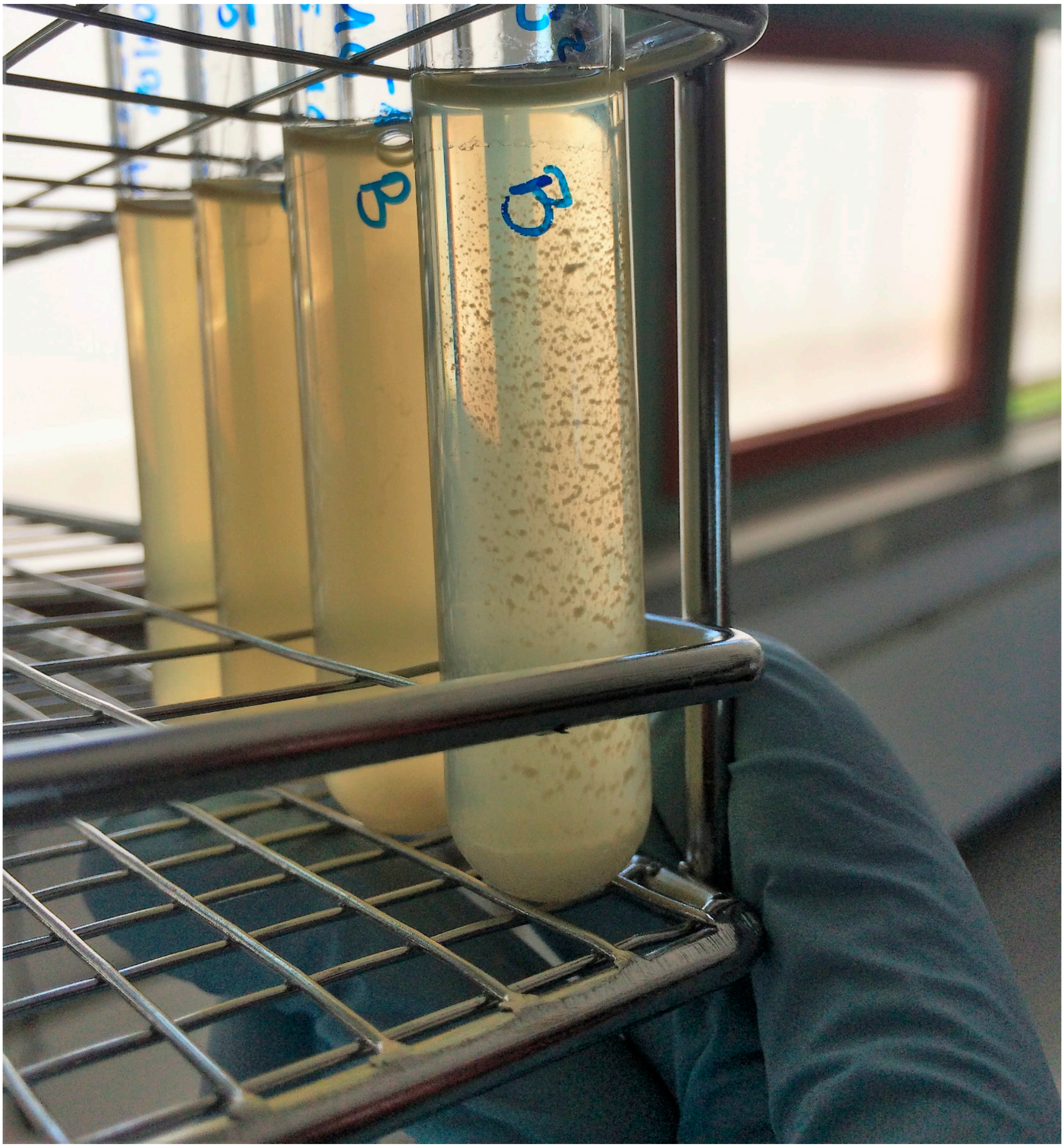

Figure S11. iVal cultivation of *P. bryantii* B$_1$4 in Hungate tubes. Cell pellet formation at the bottom of the tube and aggregate formation at the wall of the tube. Cells in other cultivation conditions are homogenously distributed.

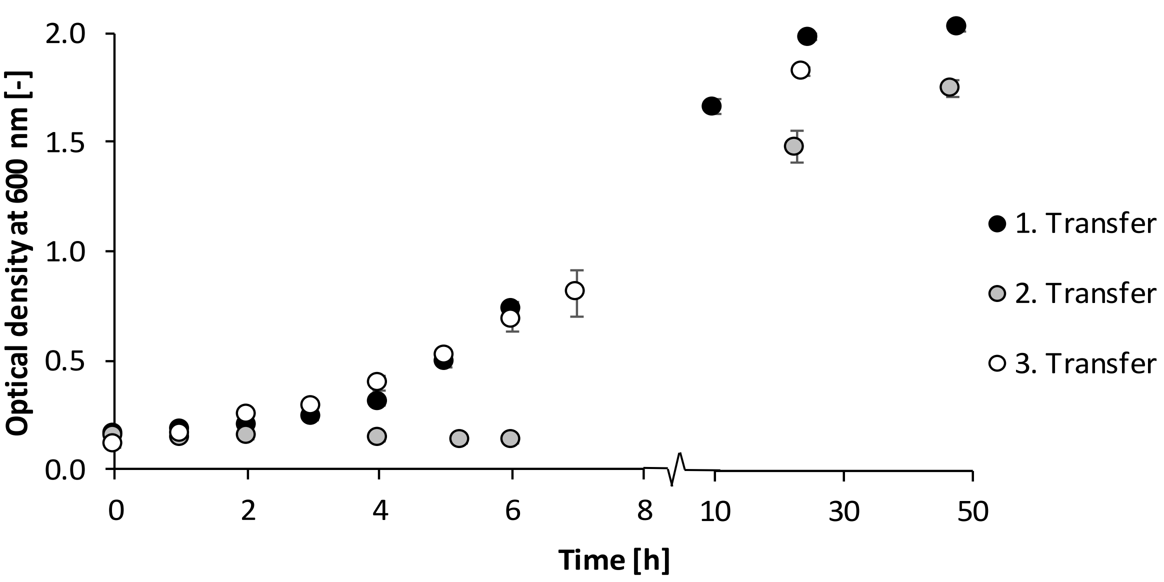

Figure S12. Growth curve of cultures transferred into SCFA-free media.
Media composition was similar except the addition of any SCFAs. Inoculation and transfer volume: 4% (v/v). Optical density measurements were conducted in Hungate tubes at a wavelength of 600 nm. Time axis is compressed from 10 to 50 h after inoculation.

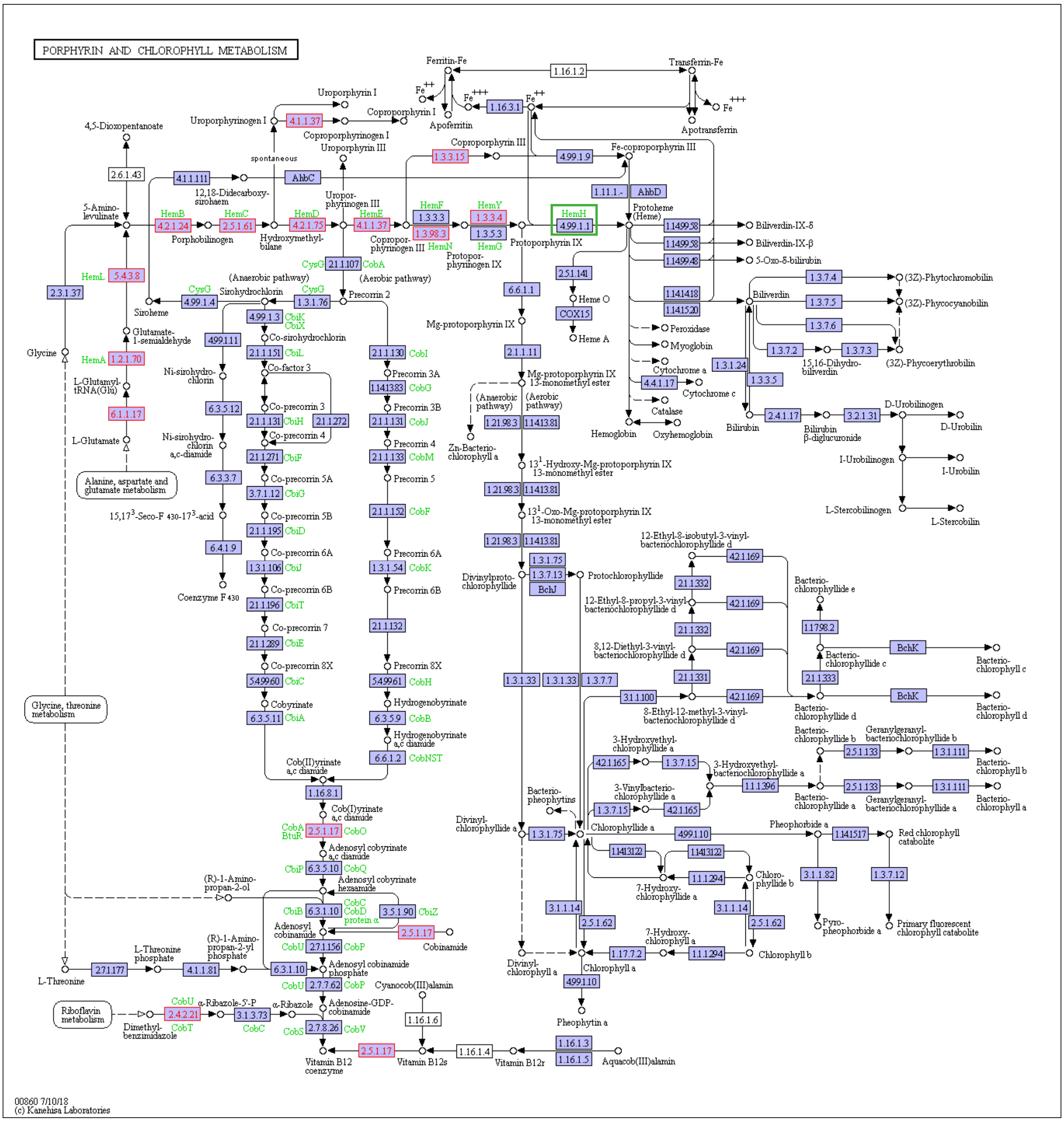

Figure S13. KEGG pathway of porphyrin and chlorophyll metabolism found in all cultivation conditions. The fields with red letters represent the found enzymes from all cultivation conditions combined. The pathway is completed from L-glutamate down to protoporphyrin IX. The orange marked enzyme is the one which annotation is missing to form heme.
